# Identification of new SdiA regulon members of *Escherichia coli*, *Enterobacter cloacae*, and *Salmonella enterica* serovars Typhimurium and Typhi

**DOI:** 10.1128/spectrum.01929-24

**Published:** 2024-10-22

**Authors:** Andrew Schwieters, Brian M. M. Ahmer

**Affiliations:** 1 Department of Microbiology, The Ohio State University, Columbus, Ohio, USA; 2 Department of Microbial Infection and Immunity, The Ohio State University, Columbus, Ohio, USA; The Hebrew University of Jerusalem, Rehovot, Israel

**Keywords:** *Salmonella*, quorum sensing, SdiA, *Enterobacter cloacae*, *Escherichia coli*

## Abstract

Bacteria can coordinate behavior in response to population density through the production, release, and detection of small molecules, a phenomenon known as quorum sensing. *Salmonella enterica* is among a group of Enterobacteriaceae that can detect signaling molecules of the *N*-acyl homoserine lactone (AHL) type but lack the ability to produce them. The AHLs are detected by the LuxR-type transcription factor, SdiA. This enables a behavior known as eavesdropping, where organisms can sense the signaling molecules of other species of bacteria. The role of SdiA remains largely unknown. Here, we use RNA-seq to more completely identify the *sdiA* regulons of two clinically significant serovars of *Salmonella enterica*: Typhimurium and Typhi. We find that their *sdiA* regulons are largely conserved despite the significant differences in pathogenic strategy and host range of these two serovars. Previous studies identified *sdiA*-regulated genes in *Escherichia coli* and *Enterobacter cloacae*, but there is surprisingly no overlap in regulon membership between the different species. This led us to individually test orthologs of each regulon member in the other species and determine that there is indeed some overlap. Unfortunately, the functions of most *sdiA*-regulated genes are unknown, with the overall function of eavesdropping in these organisms remaining unclear.

**IMPORTANCE:**

Many bacterial species detect their own population density through the production, release, and detection of small molecules (quorum sensing). *Salmonella* and other Enterobacteriaceae have a modified system that detects the *N*-acyl-homoserine lactones of other bacteria through the solo quorum sensing receptor SdiA, a behavior known as eavesdropping. The roles of *sdiA*-dependent eavesdropping in the lifecycles of these bacteria are unknown. In this study, we identify *sdiA*-dependent transcriptional responses in two clinically relevant serovars of *Salmonella*, Typhimurium and Typhi, and note that their responses are partially conserved. We also demonstrate for the first time that *sdiA*-dependent regulation of genes is partially conserved in *Enterobacter cloacae* and *Escherichia coli* as well, indicating a degree of commonality in eavesdropping among the Enterobacteriaceae.

## INTRODUCTION

**Fig 1:**
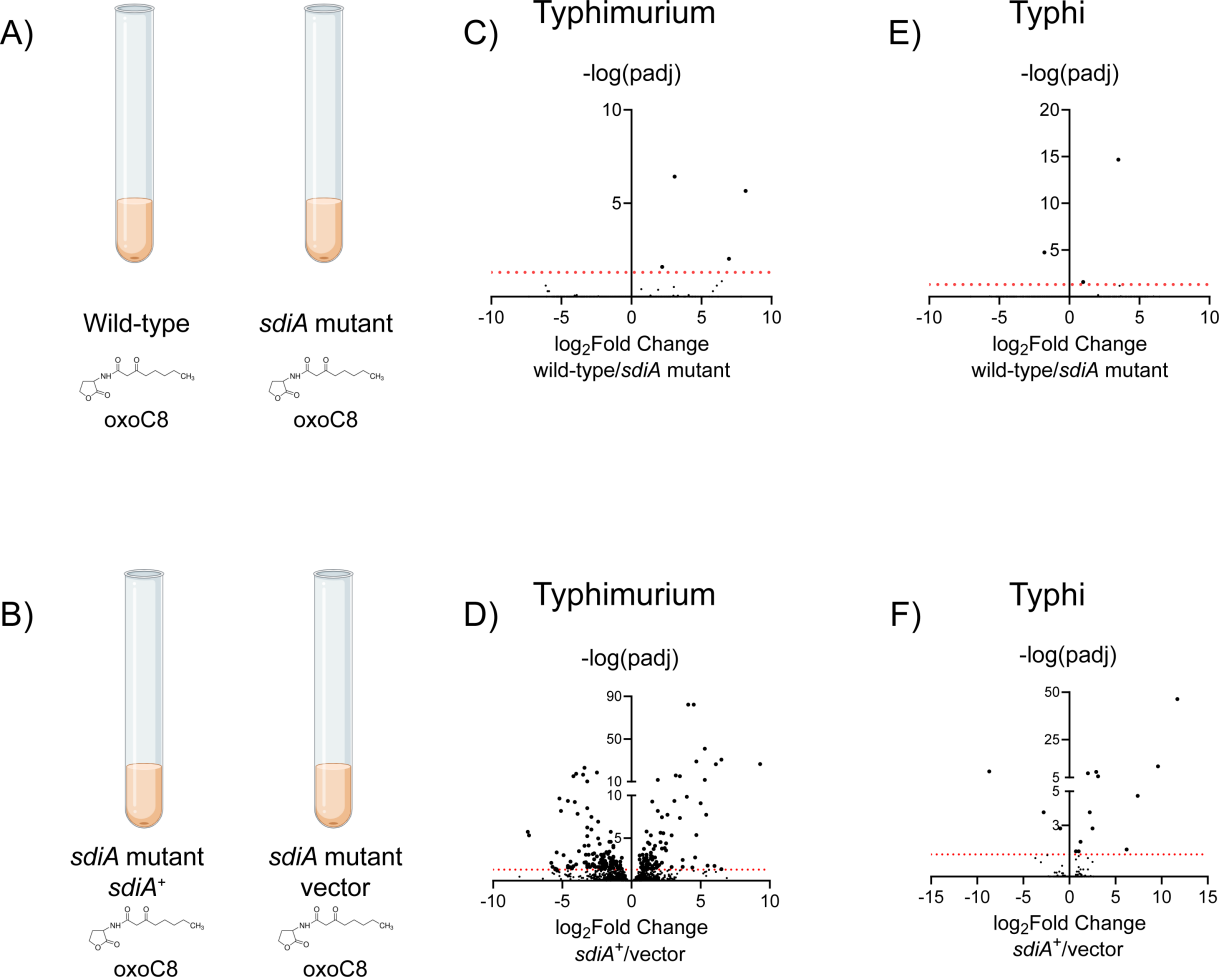
RNA-seq of *Salmonella enterica* serovars Typhimurium and Typhi to identify putative *sdiA-*regulated genes. (A) RNA was isolated from serovar Typhimurium wild-type strain 14028 or *sdiA* mutant BA612, or serovar Typhi strain Ty2 or *sdiA* mutant AMS001. All strains were grown in Lysogeny Broth (LB) with 1 µM AHL (oxoC8) to late exponential phase. (B) RNA was isolated from serovar Typhimurium strain BA612 + pJVR2 (*sdiA*+), which is an *sdiA* mutant expressing *sdiA*^Typhimurium^ from the P*araBAD* promoter, or the vector control strain, BA612 + pBAD33, or from serovar Typhi strain AMS002 + pAMS130 (*sdiA*+), which is an *sdiA* mutant expressing *sdiA*^Typhi^ from the P*araBAD* promoter, or the vector control strain AMS002 + pBAD33. All strains were grown in LB with 1 µM AHL (oxoC8) and arabinose (0.2%) to late exponential phase. (C–F) Volcano plots of gene expression differences between wild-type and *sdiA* mutant strains described in panel A (C, E), or between strains described in panel B (D, F). Each dot represents one gene. X-axes are log_2_ fold change in gene expression (wild-type/*sdiA* mutant or *sdiA^+^*/vector control) and Y-axes are -log_10_ of *P*-values (padj). Red line indicates *P* = 0.05. See Tables S3 to S6 for values of specific genes. Figure 1A and B were designed in Biorender.

Quorum sensing (QS) is a bacterial strategy of coordinating behavior based on population density through the production, release, and detection of small molecules (1). In this study, we refer specifically to QS that utilizes the detection of *N*-acyl-homoserine lactones (AHLs) by transcription factors of the LuxR type (2). A complete QS circuit of this type includes an AHL synthase of the LuxI or LuxM type and a corresponding AHL receptor of the LuxR type. AHLs can differ based on acyl chain length (4 to 18 carbons) and acyl chain differences including the degree of saturation, and the presence of hydroxyl or ketone groups (3). AHL nomenclature is based on these characteristics (e.g., N-(3-Oxooctanoyl)-L-homoserine lactone or oxoC8, shown in Fig. 1A). Each LuxR/LuxI pair synthesizes and responds to a single (or a few closely related) type of AHLs, providing a degree of species specificity. Within a confined space, or in a space with low diffusion, the AHLs accumulate to a threshold concentration and are detected by a LuxR-type transcription factor (4–7). LuxR family members often regulate genes that affect fitness when a species is at a high population density (8). Numerous QS-regulated phenotypes have been described in bacteria, including bioluminescence in *Vibrio fischeri* (9) and virulence in *Pseudomonas aeruginosa* (10).

A subset of Enterobacteriaceae encode a LuxR homolog named SdiA (11). The evolutionary history of SdiA appears to have begun as a LuxR/LuxI pair. The *Erwinia* and *Pantoea* still encode this pair where it is called ExpR/ExpI and PhzR/PhzI, respectively (11). The LuxI homolog is absent in the *Escherichia*, *Shigella*, *Salmonella*, *Klebsiella*, *Enterobacter*, *Citrobacter*, and *Cronobacter*, leaving SdiA as a LuxR solo (11, 12). Without the cognate signal synthase, SdiA detects the AHLs produced by other bacterial species (13, 14), a phenomenon referred to as eavesdropping (15, 16). Interestingly, *sdiA* has not been lost in any lineage suggesting a function important to all these organisms despite their differing environmental niches.

The role of SdiA-mediated eavesdropping remains unknown. One key piece of information to understanding this behavior is the environment in which SdiA is relevant. The genera encoding *sdiA* include many notable gastrointestinal residents and, consequently, the gut has been the environment most tested (17–19). The possibility of quorum sensing in the gut was recently reviewed (20). To briefly summarize, AHLs have been detected in both the gut and feces at low concentrations (21–23), but bioinformatic searches find few to no AHL synthases in the gut microbiome (23, 24). Although the reported concentrations of AHLs are near the detection limit of SdiA [low nanomolar (13, 25)], microenvironments in the gut could have higher concentrations (6). The implication of these findings for eavesdropping is complicated by other factors including antagonistic compounds in the gut (e.g., indole), quorum quenching activity (e.g. lactonases), and compositional shifts during infection (26–31). To determine if SdiA becomes active during bacterial transit through the gut, a reporter of SdiA activity was constructed in which *Salmonella* heritably deletes an antibiotic resistance marker from its chromosome in the presence of AHLs (18, 32). This reporter was inactive when *Salmonella* transited the gastrointestinal tract of an individual guinea pig, rabbit, and cow as well as several mice and chickens, indicating an absence of AHLs or a concentration below its detection threshold in these animals (17, 18, 33). However, the reporter strain does indicate SdiA activity when mice are concurrently infected with *Yersinia enterocolitica*, an organism known to produce AHLs (17). The reporter strain also indicates activity during transit through the gastrointestinal tract of turtles (likely due to the presence of *Aeromonas hydrophila*, a known AHL producer) (18). However, the *sdiA* mutant of *Salmonella enterica* serovar Typhimurium has no fitness defect during transit through any of these scenarios even when SdiA is active (17, 18). Thus, it is unclear if these are scenarios in which SdiA is relevant.

Another way to determine the function of SdiA is to identify the genes it regulates. Genetic screens for *sdiA*-regulated fusions have been performed in three genera: *Escherichia*, *Enterobacter*, and *Salmonella* (14, 34–36). Each screen tested ~10,000 transposon-based fusions which is roughly 68% coverage of the genome, so currently unknown regulon members may reside within the remaining 32%, or among essential genes. Microarrays and RNA-seq have also been used to identify *sdiA*-regulated genes in *Escherichia* and *Cronobacter* (19, 37). Very few genes have been tested for direct binding by SdiA so their regulons likely include direct and indirect effects (we use *sdiA* regulon throughout, rather than SdiA regulon, to emphasize this). In *Salmonella*, *sdiA* regulates two loci: the *pefI-srgD-srgA-srgB-rck-srgC* operon (hereafter referred to as the *pefI-srgC* operon) and *srgE* (14, 38–40). The *pefI-srgC* operon is known to be directly regulated by SdiA while *srgE* has not yet been tested (41). PefI and SrgA are involved in expression of Pef fimbriae through their roles as a transcriptional regulator and in the post-translational maturation of PefA, respectively (42–44). SrgB, a putative lipoprotein, and SrgC, a transcriptional regulator, have yet to be characterized. PefI and/or SrgD are involved in the regulation of flagellar motility, although mutation of *sdiA* has no effect on motility in *Salmonella,* regardless of the presence of AHLs (45–47). Rck mediates invasion of host cells by binding to epidermal growth factor receptor (48–53). SrgE is an effector protein of unknown function that is injected into host cells using the type 3 secretion system encoded within *Salmonella* pathogenicity island 2 (54). In *Escherichia coli*, *sdiA* regulates the acid fitness island, flagellar motility, prophage induction, and the virulence regulator, *ler* (19, 35, 55–60). Ler is reported to be directly regulated by SdiA (19, 56, 57). In *Enterobacter cloacae*, mutation of *sdiA* affects a collection of genes encoding hypothetical proteins along with a putative type 6 secretion system, copper transporter (CopA), O-antigen chain length determinant (FepE), and phage integrase (34). Interestingly, there is no overlap between the SdiA regulons of these three genera. The conservation of SdiA and eavesdropping represents an interesting aspect of evolution. The ligand for SdiA is externally sourced, thus limiting its activity to environments containing AHL synthesizing microbiota at sufficient population density. Despite this, *sdiA* has survived multiple speciation events spanning millions of years while maintaining completely unique regulons with no clearly related functions (14, 34, 35, 61). This paradox of simultaneous conservation and diversification across a large time frame remains one of many unsolved mysteries on the nature of SdiA and eavesdropping.

In the last 50,000 years, a serovar of *Salmonella enterica*, Typhi, has emerged and is currently undergoing reductive evolution as its host range becomes restricted to humans (62). Serovars Typhi and Typhimurium have significant differences in their pathogenic strategy. Serovar Typhimurium invades intestinal epithelial cells, inducing inflammation to eliminate competitors in the lumen (31, 63, 64). Serovar Typhi limits intestinal inflammation and replication in the lumen in favor of colonization at systemic sites (65–67). Additionally, Typhi infections can develop into a chronic carrier state through the formation of biofilms on gallstones and gallbladder epithelium (68, 69). This change in host range and pathogenesis could impart selective pressure on the response of serovar Typhi to foreign AHLs, yet the *sdiA* regulon of Typhi has not been investigated.

We sought to identify the regulons of *sdiA* more thoroughly and determine what effect, if any, the reduction in host range has had on serovar Typhi’s transcriptional responses to foreign AHLs. Using RNA-seq, we measured the *sdiA-*dependent response of serovars Typhimurium and Typhi to AHLs. Differentially regulated genes were validated via the construction and testing of transcriptional fusions, revealing regulons comprising six loci in Typhimurium and five in Typhi with four common to both. Other genes were identified that respond to plasmid-based expression of *sdiA*, but these could not be validated using *sdiA* expressed from its native position in the chromosome. These may be artifactual or require additional unknown stimuli for expression. Additionally, we constructed fusions to orthologs of genes in *Salmonella*, *E. coli*, and *E. cloacae* that were known to be regulated by *sdiA* in one genus but not the others. No new regulon members in *Salmonella* were discovered by this approach, but one new regulon member was found in *E. coli* and three new regulon members were found in *E. cloacae*.

## RESULTS

### Identification of SdiA-regulated loci in Typhimurium and Typhi

A genetic screen previously revealed seven members of the *Salmonella enterica* serovar Typhimurium (*S*. Typhimurium) SdiA regulon encoded in two loci: the *srgE* gene located in the chromosome and the *pefI-srgD-srgA-srgB-rck-srgC* (*pefI-srgC*) operon located on the virulence plasmid pSLT (13, 14). The *sdiA* regulon of *Salmonella enterica* serovar Typhi (*S*. Typhi) has never been investigated. To identify additional regulon members, we performed RNA-seq. Unfortunately, *sdiA*-dependent regulation is weak in broth culture and higher in motility agar due to its direct regulation by alternative sigma factor FliA (47, 70). Other regulators of the *sdiA* promoter have been described, including Crp and LeuO (71). RNA for downstream analysis of *sdiA*-dependent changes would preferably be sourced from bacteria grown in motility agar, but we have not been able to isolate quality RNA from bacteria grown in this manner. Therefore, we isolated RNA from wild-type and *sdiA* mutant bacteria grown in liquid culture containing AHL [oxoC8 is the optimal AHL for SdiA activation (13) and was the sole AHL used in this study]. In a second experiment, RNA was isolated from *sdiA* mutant strains containing *sdiA* under the control of an arabinose-inducible promoter on plasmid pBAD18, compared to a vector control, also in liquid culture containing AHL (Fig. 1). After processing, sequencing, and analysis, we observed a small number of differentially expressed genes in both serovars when expressing *sdiA* from its native position in the chromosome (Fig. 1C and E). Plasmid-based expression of *sdiA* increased the number of differentially regulated genes (Fig. 1D and F). Results from all four RNA-seq experiments can be found in Tables S3 to S6.

To validate the RNA-seq results, we constructed transcriptional fusions to each differentially regulated gene (defined as fold change greater than 4 and *P*-value ≤0.05). Each putative *sdiA*-regulated promoter was cloned upstream of the *luxCDABE* operon of plasmid pSB401 or had already been constructed in previous studies (see Table S1) (72). In some cases, multiple regions were cloned for a single locus as it was not always clear where the promoter might be. Additionally, if a gene was differentially regulated in one serovar but an ortholog is present in the other serovar, reporters were constructed for both serovars. This turned out to be a wise choice as some regulon members were identified in this manner.

For *S*. Typhimurium, we tested approximately 60 reporters representing 50 loci. For *S*. Typhi, we tested 15 reporters representing 15 loci. Each reporter was placed into wild-type and *sdiA* mutant strains of the relevant serovar. Some were also placed into strains with plasmid-encoded *sdiA* (*sdiA* mutant strains containing *sdiA* under the control of an arabinose-inducible promoter on plasmid pBAD18). Luciferase activity of these strains was measured over time in the presence or absence of 1 µM AHL. Some fusions were regulated by plasmid-encoded but not chromosome-encoded *sdiA* (Fig. S1) while others were regulated under neither condition (Fig. S2). Here, genes were only “confirmed” as *sdiA*-regulated if they respond to *sdiA* expressed from its native position in the chromosome. In total, four loci are regulated by *sdiA* in both serovars, two are exclusively regulated by *sdiA* in *S*. Typhimurium (totaling six), and one is exclusively regulated by *sdiA* in *S*. Typhi (totaling five). Genes of limited characterization were renamed to *srg* (*sdiA*-regulated gene) and are described below. For each reporter, we calculated the maximum fold activation (wild-type vs *sdiA* mutant) in both motility agar and broth with or without AHL. In motility agar, raw luciferase values are shown while broth culture luciferase readings were normalized to growth (OD_600_) at that time point (neither serovar’s *sdiA* mutant exhibits growth or motility defects). These values are shown in Table 1.

**TABLE 1:** *sdiA*-dependent regulation of transcriptional fusions in *S. enterica*, *E. coli*, and *E.cloacae*

­			Motility agar^*a*^		LB^*a*^	
Species/Serovar	Gene(s)	Plasmid	AHL^*b*^	Solvent^*c*^	AHL^*b*^	Solvent^*c*^
*Salmonella enterica* serovar Typhimurium	*srgE*	pJNS25	19	2. 3	5.2	1.2
	*srgF*	pAMS148	5.0	1.6	1.6	1.5
	*srgGH*	pAMS145	4.5	2.2	2.1	1.6
	*srgKJ*	pJLD202	3.0	1.3	1.6	1.5
	*menFDHBCE*	pAMS291	1.8	1.1	1.6	1.8
	*pefI-srgC*	pBA428	8.2	1.3	4.9	2.5
						
*Salmonella enterica* serovar Typhi	*srgIL*	pAMS201	14	2.3	1.7	1.4
	*srgF*	pAMS205	6. 3	3.7	3.2	2.0
	*srgGH*	pAMS265	2.5	1.0	4.4	1.1
	*srgKJ*	pAMS050	2.8	1.1	1.5	1.1
	*menFDHBCE*	pAMS202	2.7	1.2	1.5	1.4
	*srgE* ^Typhimurium^	pJNS25	ND^*d*^	ND^*d*^	2.8	2.1
						
*E. coli*	*fepE*	pAMS366	ND^*d*^	ND^*d*^	−7.3	−8.0
						
*E. cloacae*	*srgKJ*	pAMS228	ND^*d*^	ND^*d*^	1.9	1.7
	*menFDHBCE*	pAMS362	ND^*d*^	ND^*d*^	−105	−70
	*srgG*	pAMS368	ND^*d*^	ND^*d*^	4.2	3.6

^*a*^ Value is the largest fold change in *sdiA*-dependent expression of each fusion throughout the time course in the media of each column (kinetics shown in Fig. 2 and 3) . Positive values indicate an *sdiA*-dependent increase in expression, while negative values indicate *sdiA*-dependent decrease in expression.

^*b*^ The AHL is 1 µM oxoC8.

^*c*^ The solvent control is 0.1% ethyl acetate.

^*d*^ ND, not determined.

**Fig 2:**
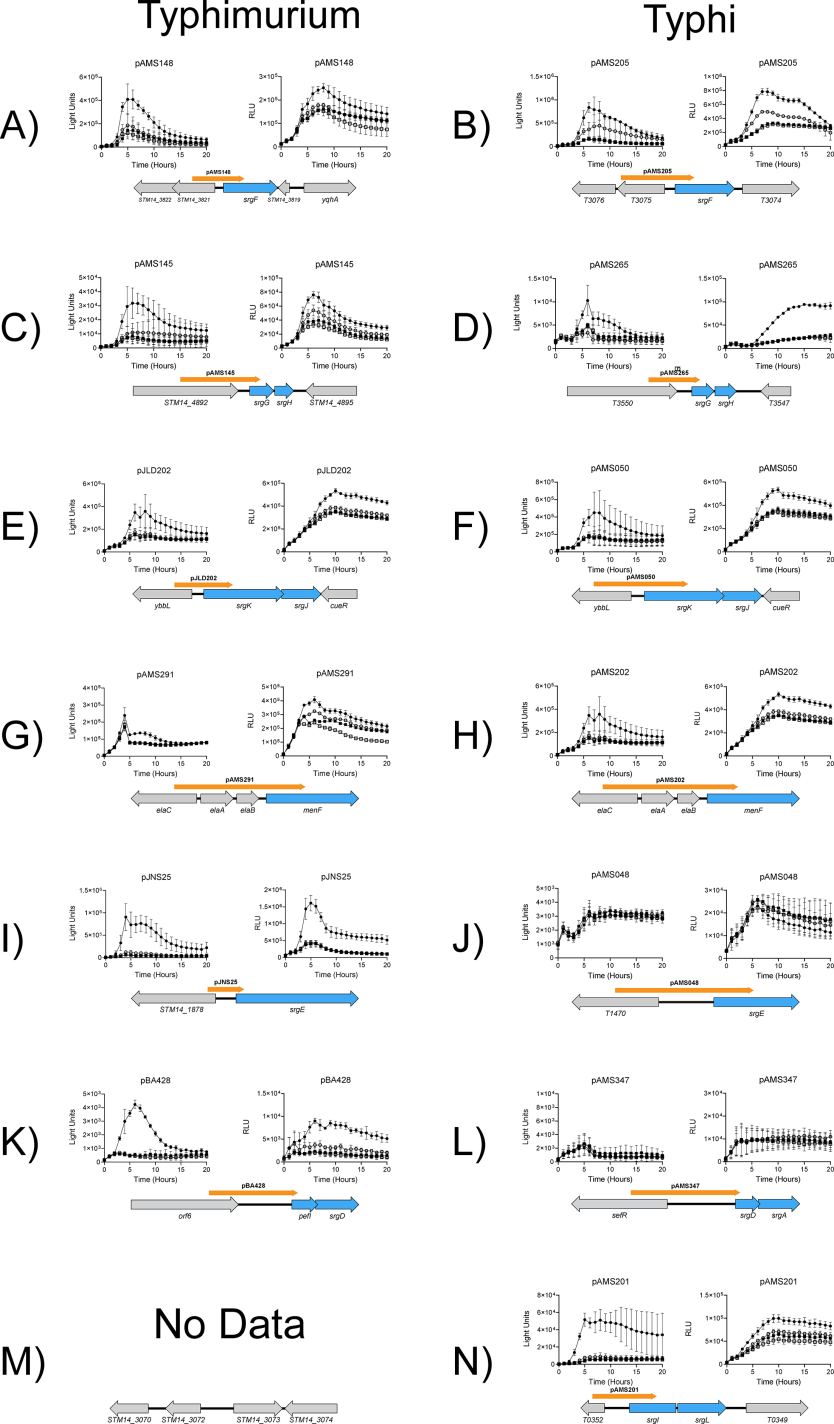
Validation of *sdiA*-regulated genes in serovars Typhimurium and Typhi. Dependence of luciferase reporters on *sdiA* and AHL. Each reporter was tested in motility agar (left graph) and LB (right graph) for luciferase activity in wild-type (circles) and *sdiA* mutant (squares) backgrounds. Each media was supplemented with either AHL (oxoc8) at 1 µM (closed symbols) or solvent (ethyl acetate) at 0.1% vol/vol (open symbols). Diagrams of genes identified using RNA-seq (in blue) and their genomic context (not to scale) are shown under their corresponding luciferase data. The cloned promoter is displayed as an orange arrow. Graphs 2A, C, E, G, I, K, and M show data in *S*. Typhimurium whose wild-type is 14028, *sdiA* mutant is BA612. Graphs 2B, D, F, H, J, L, and N show data in *S*. Typhi, whose wild-type is Ty2 and *sdiA* mutant is AMS002. In each graph, X-axis is time (in hours). Y-axis is either raw luciferase activity (motility agar) or luciferase activity normalized to growth (OD_600_) at the corresponding time point. Each time point represents the mean ± standard deviation of nine replicates (3 technical × 3 biological replicates).

**Fig 3:**
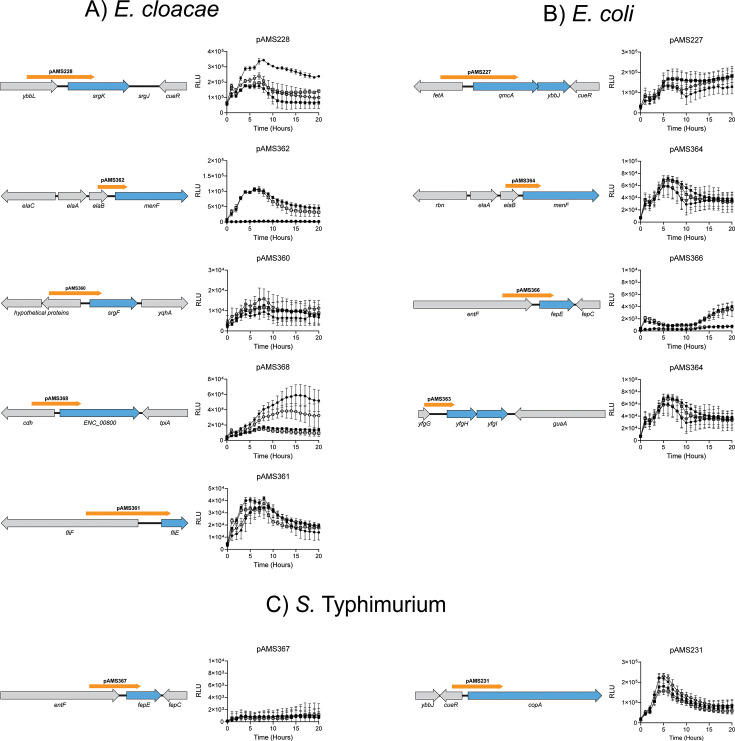
Cross-species validation of *sdiA*-regulated genes in *Salmonella*, *E. coli*, and *E. cloacae*. Dependence of luciferase reporters on *sdiA* and AHL. Each reporter was tested in LB (right graph) for luciferase activity in wild-type (circles) and *sdiA* mutant (squares) backgrounds. Each media was supplemented with either AHL (oxoc8) at 1 µM (closed symbols) or solvent (EA) at 0.1% vol/vol (open symbols). In each graph, X-axis is time (in hours). Y-axis is luciferase activity normalized to growth (OD_600_) at the corresponding time point. Diagrams of genes of interest (in blue) and their genomic context (not to scale) are shown under their corresponding luciferase data. The cloned promoter is displayed as an orange arrow. Figure 2A indicates promoters and activity in *E. cloacae*, whose wild-type is JLD401 and *sdiA* mutant is ASD401. Figure 2B is *E. coli*, whose wild-type is MG1655 and *sdiA* mutant is JNS21. Figure 2C is *S*. Typhimurium, whose wild-type is 14028 and *sdiA* mutant is BA612. Each time point represents the mean ± SD of nine replicates (3 technical × 3 biological replicates).

### SrgF, SrgGH, SrgKJ, and MenFDHBCE are regulated by SdiA in both Typhimurium and Typhi

The first newly identified gene, *srgF* (*STM14_3820* in Typhimurium and *T06040* in Typhi), was identified in the RNA-seq results as upregulated by plasmid-based expression of *sdiA* in both serovars, but not in either RNA-seq experiment using endogenous expression of *sdiA*. Reporter constructs of the *srgF* promoter were generated for each serovar and placed into wild-type and *sdiA* mutant strains. These strains were grown in the presence or absence of AHL in LB broth or motility agar and luciferase activity was recorded over time. AHL increased the activity of the reporter in an *sdiA*-dependent manner in both media, with higher activation in motility agar (Fig. 2A). The *srgF* reporter of *S*. Typhi behaved similarly (Fig. 2B). As observed with other regulon members, there is a small amount of *sdiA*-dependent but AHL-independent regulation. SrgF is annotated as a putative ATP-dependent RNA helicase-like protein in serovar Typhimurium. We examined SrgF using bioinformatic tools HHPred and FoldSeek, which found no similarity to previously identified protein domains (73, 74). Phobius identified a possible transmembrane domain in the first 30 residues and cytoplasmic orientation of the remaining protein (75). Literature searches for SrgF revealed occasional hits in genetic screens involving colonization of chickens (76), motility (77, 78), aquatic survival (79), and phage infection (80). We have previously observed no effect of AHL or *sdiA* on motility (47). We tested an *srgF* mutant of *S*. Typhimurium for fitness in mice rendered susceptible to gastroenteritis by a high-fat diet (81) and found the mutant to have little or no phenotype (Fig. S3A). Interestingly, transcriptomic studies indicate a significant amount of basal expression of *srgF* during *in vitro* growth, especially compared to other regulon members (82, 83). The role of this gene in *Salmonella* requires additional study.

The second locus, which we refer to as *srgGH (STM14_4893–4894*), was identified by RNA-seq of *Typhimurium* expressing *sdiA* from a plasmid. The P*_srgGH_* reporter (pAMS145) exhibits both *sdiA* and AHL-dependent activation in motility agar and LB (Fig. 2C). Although not identified in either *S*. Typhi RNA-seq experiment, a promoter fusion of the *S*. Typhi *srgGH* orthologs (pAMS265) is also regulated (Fig. 2D). The *srgG* and *srgH* genes appear to be remnants of functional genes present in other bacterial species. SrgG encodes a 55 amino acid fragment of the N-terminus of a putative citrate transporter in *Salmonella bongori* (SBG_RS18665) and *Enterobacter cloacae* (this gene is *sdiA*-regulated, see below). SrgH is a 44 amino acid fragment homologous to the C-terminal domain of *ushB (cdh* in *E. coli*). UshB is non-functional in *Salmonella* (84), and no published literature on either SrgG or SrgH was found. A mutant of *S*. Typhimurium lacking *srgH* has no fitness defect during gastrointestinal infection of mice (Fig. S3A).

SrgKJ (*ybbKJ* in Typhimurium and *T2359-2360* in Typhi) was identified in three RNA-seq experiments, and both reporters (pJLD202 and pAMS050) exhibit *sdiA* and AHL-dependent regulation (Fig. 2E and F). Previous characterization of *E. coli* orthologs *qmcA-ybbJ* indicates that QmcA is likely involved in protein turnover and YbbJ acts as a helper protein (85). The protein target(s) of QmcA and its orthologs are unknown. Like *srgF, srgK* and *sdiA* were implicated in phage resistance in a recent Tn-seq study (80).

Finally, we identified the *menFDHBCE* operon. This locus was only identified by RNA-seq using plasmid-based expression of *sdiA* in *S*. Typhi but is encoded in both serovars. Reporters pAMS291 and pAMS202 show weak regulation by *sdiA* in both serovars (Fig. 2G and H). *E. coli* and *Salmonella* encode two isochorismate synthases, which make isochorismate for synthesis of both menaquinone (*menF*) and enterobactin (*entC*) (86–88). Menaquinones have a role in respiration induced by anaerobic conditions while enterobactin is a siderophore used to acquire iron from the environment (87). It is unclear what role menaquinones may play in SdiA-mediated eavesdropping. We observed no *sdiA* or AHL-dependent regulation of *entC* or any effect of iron availability on SdiA activity (data not shown).

### Serovar-specific regulon members

A past genetic screen for *sdiA*-regulated genes in Typhimurium yielded two loci: *srgE* and *pefI-srgC* (13, 14). As previously published, the reporters for these loci (pJNS25 and pBA428) are regulated by *sdiA* and AHL (Fig. 2I and K) (13, 47). *S*. Typhi does not harbor the virulence plasmid that encodes *pefI-srgC* but does encode *srgDAB* orthologs (*T4538-4540*) in the chromosome as well as an *srgE* ortholog (54, 89). Neither constructed fusion exhibited regulation by *sdiA* (Fig. 2J and L). These two loci have been previously examined in a third serovar, Enteritidis (41, 54). The virulence plasmid of *S*. Enteritidis has lost *sdiA*-dependent regulation of its *pefI-srgC* operon and does not encode *srgE*. (41, 54). In the context of these three serovars, *sdiA*-dependent regulation of the *pefI-srgC* operon and *srgE* is an exclusive trait of *S*. Typhimurium despite the significant host range overlap of *S*. Typhimurium and *S*. Enteritidis. A competitive infection between wild-type *S*. Typhimurium and an *srgE* mutant revealed no fitness defects in a mouse model of gastrointestinal infection (Fig. S3A).

A locus encoding T0351-0350 (*srgIL*), orthologous to *yfgHI* in *E. coli*, respectively, was found by RNA-seq to be upregulated by plasmid-based expression of *sdiA* in *S*. Typhi. Orthologs are not found in Typhimurium (Fig. 2M). The reporter for *srgIL*, pAMS201, is strongly regulated by *sdiA* and AHL (Fig. 2N). The first gene of the operon, *T0351*, is annotated as a pseudogene. However, an alternative reading frame can be found within this pseudogene that produces a SlyB-like lipoprotein, the same protein family as YfgH. The original annotation may be incorrect but the expression of the SrgI protein was not confirmed here. In *E. coli*, *yfgH* is predicted to be involved in outer membrane integrity (90) while *yfgI* mutants have been shown to be susceptible to DNA damage (91). The hypothesis that SdiA could mediate resistance to DNA damage was assessed for both serovars using two stressors: nalidixic acid and ultraviolet light (UV). Inhibitory concentrations of nalidixic acid were quantified for wild-type, *sdiA* mutant, and plasmid complementation strains grown in AHL or solvent control (Fig. S3B). A difference (<2-fold) was only observed using plasmid-based expression of *sdiA*. Given that *sdiA* had much stronger effects on P*_srgIL_* in motility agar, we assessed this putative phenotype using a disk diffusion assay in motility agar, using a twofold dilution series of nalidixic acid. No differences in zones of inhibition were apparent (Fig. S3D). The results of the nalidixic acid challenge are also consistent with our previous report that *sdiA* has no effect on antibiotic resistance in *S*. Typhimurium (35). For UV-mediated DNA damage, we generated survival curves against increasing doses of UV. Differences between wild-type and *sdiA* mutant strains were never observed in either endogenous or plasmid-based *sdiA* backgrounds (Fig. S3C). Thus, we find no evidence for protection from DNA damage by *sdiA* in either serovar.

### Unconfirmed regulon members

It is worth noting that several virulence-associated loci were found to be regulated by plasmid-encoded *sdiA* but not under endogenous expression conditions, including promoters of *Salmonella* pathogenicity island 1, flagellar genes, and type 1 fimbriae (Fig. S1). The absence of regulation at the endogenous level could be artifacts from plasmid-based expression of *sdiA*, or true regulon members for which the proper environmental conditions for *sdiA*-dependent expression have not yet been found. We identified several fusions that were also differentially regulated by plasmid-encoded *sdiA* that are known to be regulated by other extrinsic elements: P*_leuA_* (pAMS173) and leucine, P*_proVWX_* (pAMS172) and osmotic stress, and P*_dpiBA_* (pAMS143) and citrate (92–94). We manipulated leucine and citrate levels as well as osmolarity of the medium. This did alter activity of the corresponding reporter, but it did not cause *sdiA*-dependent regulation at the endogenous level (data not shown). Based on RNA-seq results, a significant number of prophage genes were repressed by plasmid-based expression of *sdiA* in serovar Typhimurium. These have not yet been tested using fusions, and the potential relationship between *sdiA* and prophage elements requires further investigation.

### Cross screening SdiA regulons reveals semi-conservation between species

The published *sdiA* regulon members are different in *E. coli*, *S. enterica*, and *Enterobacter cloacae* (14, 34, 35). We hypothesized that at least a portion of the regulons are evolutionarily conserved. To test this, we identified orthologs of each regulon member in species where that ortholog is not known to be regulated by *sdiA*. We then constructed transcriptional fusions to each and tested them for *sdiA*-dependent regulation. For clarity, reporters were tested only in the species from which the promoter was amplified.

In *E. cloacae*, orthologs of four *sdiA*-regulated loci of *Salmonella enterica* were identified: *srgKJ*, *menFDHBCE*, *srgF*, and *srgG*. The *srgG* ortholog, *ENC_00800*, is full length in *E. cloacae*. One ortholog of the *E. coli sdiA* regulon was identified in *E. cloacae: fliE*.

Using constructed luciferase fusions, we find three to be regulated by *sdiA* in *E. cloacae: srgKJ*, *srgG*, and *menFDHBCE* (Fig. 3A). Regulation of P*_menFDHBCE_* is strongly *sdiA* regulated but fully AHL-independent, a trait observed in some other *E. cloacae* regulon members (34). This adds three members to the *sdiA* regulon of *E. cloacae*, all of which are the first regulon members conserved between *Salmonella* and *E. cloacae*.

**Fig 4:**
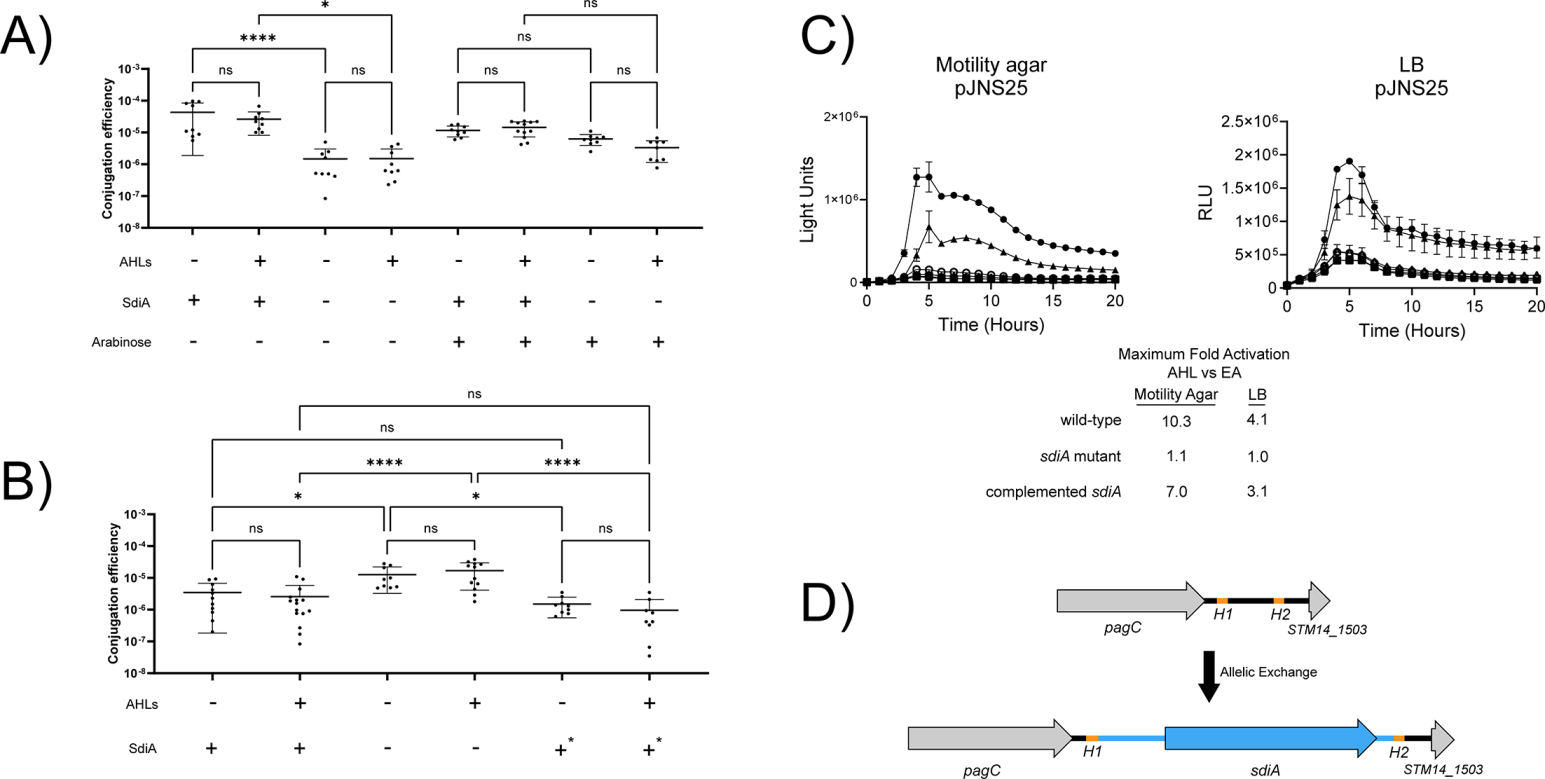
Repression of pSLT self-transmission by *sdiA*. (A, B) Conjugation efficiency (transconjugants per donor) was determined for matings between donor strains carrying pSLT*^spv^*^::MudJ^ and recipient strain BA770. Matings were performed overnight on LB agar with indicated supplements: AHL ± (oxoC8 at 1 µM or EA at 0.1% vol/vol) and ± arabinose (0.2% or none). (A) SdiA+ uses donor BA612 + pJVR2; SdiA- uses donor BA612 + pBAD33. (B) SdiA+ uses donor BA1541; SdiA- uses donor AMS171; SdiA+* donor is AMS246 (complemented *sdiA*). (C) Activity of P*srgE* reporter plasmid in wild-type 14028 (circle), *sdiA* mutant BA612 (square), and complemented *sdiA* mutant AMS203 (triangle) in motility agar or LB, + AHL (closed) or EA (open). Fold activation of each strain was calculated as the expression in AHL vs solvent. The highest value is listed in the table. (D) Graphical representation of placement of *sdiA* into the intergenic region between *pagC* and *STM14_1503* (see Materials and Methods for details of construction).

In *E. coli*, orthologs of three *sdiA*-regulated loci from *Salmonella enterica* were identified: *qmcA(ybbK)-ybbJ*, *menFDHBCE*, and *yfgHI* (Fig. 4B). One ortholog of the *E. cloacae sdiA* regulon was identified in *E. coli: fepE*. We were unable to construct a transcriptional reporter for *copA* of *E. coli*. One of the four constructed fusions is regulated by *sdiA: fepE*, and this occurs in an AHL-independent manner (Fig. 3B). The *fepE* gene is the only *sdiA* regulon member conserved between *E. coli* and *E. cloacae*.

**Fig 5:**
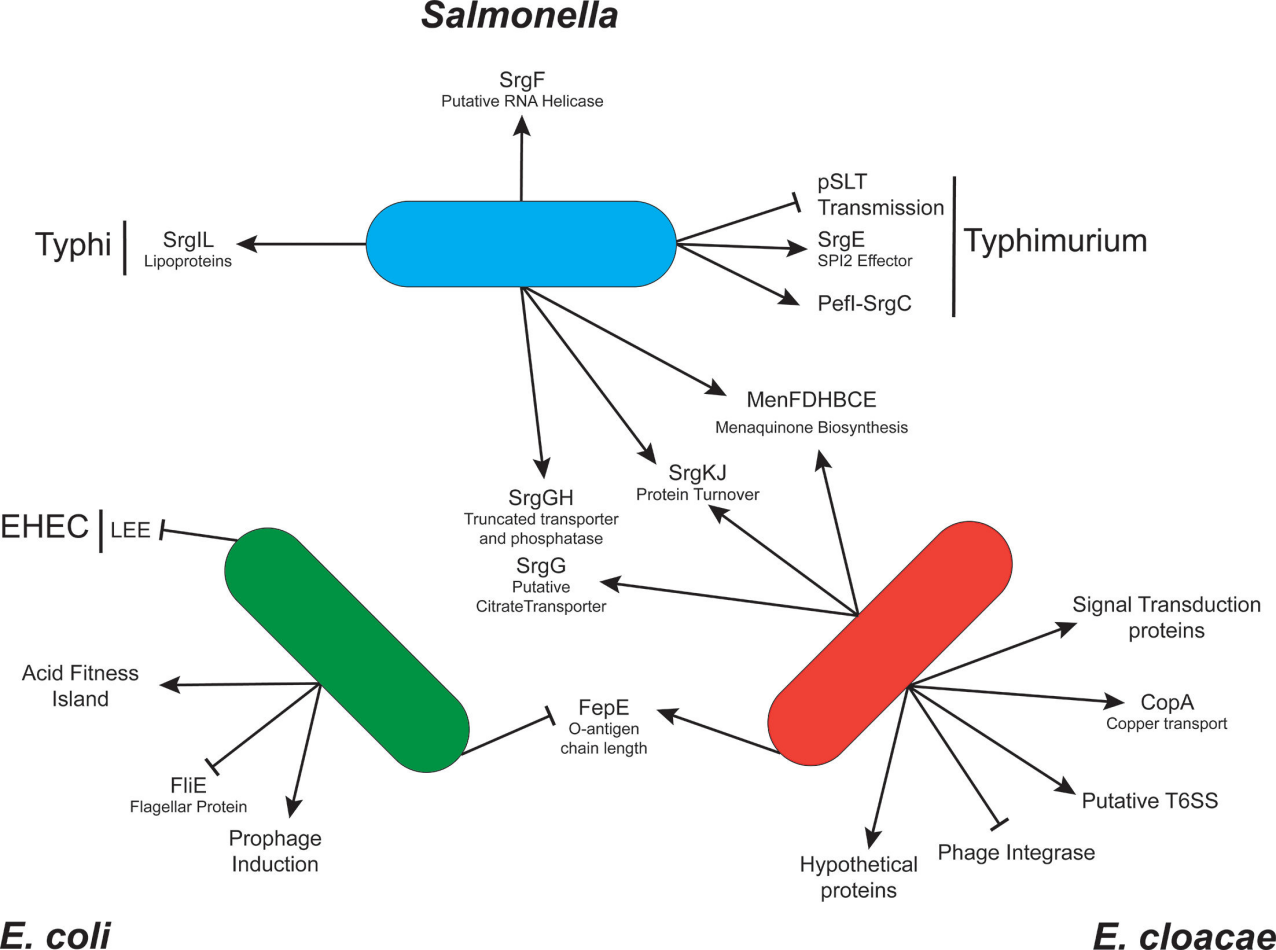
Summary of the *sdiA* regulons. Diagram of known *sdiA* regulons of *Salmonella*, *E. coli*, and *E. cloacae* based on this study and previous literature. Arrows indicate transcriptional activation or increased phenotype. Blunt arrows indicate transcriptional repression or decreased phenotype. Abbreviations: EHEC (enterohemorrhagic *E. coli*), LEE (locus of enterocyte effacement).

In *S*. Typhimurium, two orthologs of *sdiA*-regulated loci from *E. cloacae* were identified: *fepE* and *copA*. Neither were regulated by *sdiA* or AHL in either broth or motility agar (Fig. 3C, motility agar not shown). A *fliE* reporter was not tested based on the negative results of other flagella reporters (Fig. S1). The *copA* gene is regulated by CueR, whose gene is adjacent and inversely oriented (95). We tested both orientations of the reporter (i.e., measuring *copA* or *cueR* transcription) in broth and motility agar, with and without copper at stress-inducing concentrations, none of which led to AHL- or *sdiA*-dependent regulation (data not shown). The regulons of these three species as currently understood are summarized in Fig. 5.

### SdiA affects pSLT conjugation efficiency independent of AHL

A subset of *Salmonella* serovars, including Typhimurium but not Typhi, harbor IncF plasmids that range in size from 50 to 90 kb (96). The 90 kb plasmid of serovar Typhimurium, pSLT, is self-transmissible (97–99). The RNA-seq data set from plasmid-based expression of *sdiA* showed an upregulation of pSLT conjugation genes. To determine whether or not there was an effect on transmission frequency, we used a conjugation assay (97). Wild-type or *sdiA* mutant donor strains with a *spv*::Mu*d*J mutation (kan^r^) on their pSLT plasmid were mated with a recipient strain lacking pSLT (BA770, nal^r^) in the presence or absence of AHL. The frequency of kan^r^ nal^r^ transconjugants obtained per donor was three- to six-fold lower in the wild-type compared to *sdiA* mutant, suggesting that *sdiA* represses conjugation (Fig. 4B). Expression of *sdiA* from a plasmid increases conjugation frequency, though this only occurred in the absence of the inducer (arabinose) (Fig. 5A). When arabinose was provided to induce *sdiA* expression, *sdiA* no longer had any effect on conjugation efficiency (Fig. 4A). AHL had no significant effect on conjugation efficiency in either strain background.

Since plasmid-based expression of *sdiA* gave different effects on conjugation frequency than expression of *sdiA* from its native position in the chromosome, we complemented the chromosomal *sdiA* mutation with a functional copy of *sdiA* inserted at a neutral location in the chromosome located downstream of *pagC* (strain AMS203, Fig. 4D) (100). To confirm that this strain restored *sdiA* activity, we measured luciferase activity from the P*_srgE_* reporter plasmid pJNS25 and observed complementation of *sdiA* function (Fig. 4C). Conjugation efficiency is restored to wild-type levels by this method of complementation (Fig. 4B). We conclude from these findings that *sdiA* has a small negative effect on the frequency of pSLT transmission and AHLs do not alter this phenotype. The mechanism(s) by which *sdiA* regulates plasmid transmission and reasons for the confounding effects of plasmid-based expression and arabinose are unclear at this point. It should be noted that the recipient, BA770, encodes *sdiA*. We did not determine if *sdiA* can affect conjugation efficiency as a recipient, but a previous study suggests that *sdiA* can repress plasmid transmission between a donor *Pseudomonas aeruginosa* and recipient *E. coli* (101).

## DISCUSSION

Quorum sensing is a strategy used by bacteria to coordinate behavior within a species upon reaching a population density threshold. Bacteria have evolved to link a diverse array of behaviors to population density, including competence, virulence, biofilm formation, bioluminescence, and phage defense (102–107). A subset of Enterobacteriaceae, including model organisms like *Salmonella* and *E. coli*, have lost their signal synthase to facilitate an alternative behavior: eavesdropping (11). The LuxR solo SdiA detects foreign AHLs, preferably with acyl chain lengths of 6 or 8 and a ketone modification on the third carbon (oxoC6, oxoC8) (47). Experimentally, SdiA has been shown to detect the AHLs produced by a wide range of genera, including *Agrobacterium*, *Aeromonas, Hafnia, Pantoeae, Pectobacterium,* and *Yersinia* (17, 18, 33, 47). However, *sdiA* mutants have almost no reported *in vivo* defects (even when AHLs are present), leaving it unclear in which scenarios *sdiA*-mediated eavesdropping is relevant. Additionally, the body of literature on phenotypes is complex and ultimately inconclusive on what exactly these bacteria do differently when they detect foreign AHLs.

The *sdiA* regulon of *Salmonella enterica* has only been studied in one serovar, Typhimurium, and only using a genetic screen that was 68% saturated (14). That study identified two *sdiA*-regulated loci: the *pefI-srgC* operon and *srgE* (13, 14). Here, we investigated the *sdiA*-dependent transcriptional responses of this same organism using RNA-seq, allowing for full coverage of the genome. An identical experiment was performed using serovar Typhi, representing an interesting contrast as a host-adapted serovar (66). *Salmonella* SdiA is most active in motility agar (because FliA directly regulates the *sdiA* promoter) (47, 70). As we have been unable to isolate quality RNA in semi-solid media, we instead collected RNA from wild-type and *sdiA* mutants grown in LB. Additionally, RNA was collected from strains expressing *sdiA* on a plasmid, substantially increasing its activity and the number of differentially expressed genes. Over 200 potential members of the *Salmonella sdiA* regulon in serovars Typhimurium and Typhi were found, mostly from plasmid-based expression of *sdiA* in serovar Typhimurium. Increasing the copy number of *sdiA* on a plasmid is a commonly used approach as it bypasses the need for AHL entirely (13). This is also quite risky given the propensity for phenotypes that occur in plasmid-based expression backgrounds to disappear under endogenous expression conditions (e.g., multiple drug resistance and mini-cell formation) (35, 108, 109). Using transcriptional fusions, we tested almost all of the genes identified using plasmid-based expression of *sdiA*. Most were confirmed to be regulated by *sdiA* expressed from a plasmid but not by *sdiA* expressed from its native position in the chromosome (Fig. S1). However, in this study, we considered a gene to be a verified member of the *sdiA* regulon only if the gene has been confirmed to be regulated by *sdiA* expressed from its native position in the chromosome. This greatly limits the size of the regulon and is likely excluding real members. It is probable that our *in vitro* growth conditions are not permissive for expression of some of the regulon members. Thus, the list of genes that respond to plasmid-based *sdiA* should not be dismissed entirely. It should also be noted that we have not yet determined which genes are directly regulated by SdiA and which are indirect (which is why we refer to the *sdiA* regulon rather than the SdiA regulon). Thus, our current understanding of the regulons of these different species includes the direct and indirect effects.

In serovar Typhimurium, the regulon includes four new loci (six total). In serovar Typhi, five loci are *sdiA* regulated (all newly discovered here), four of which are shared with serovar Typhimurium (Fig. 3). The first two regulon members identified in Typhimurium, *pefI-srgC* and *srgE*, were not regulated in serovar Typhi (14). Another broad host range non-typhoidal serovar, Enteritidis, has lost the SdiA-specific promoter of its *pefI-srgC* operon and does not encode *srgE* at all (41, 54). Host-range reduction alone therefore may not be sufficient to explain this change in regulon membership. In addition, *sdiA* regulates transmission of the virulence plasmid, pSLT, independently of AHL. Transmission is known to be regulated by multiple factors, including nutrient availability, osmolarity, and microaerophilic conditions and occurs both *in vitro* and *in vivo* (97, 99, 110). SrgIL is the single Typhi-specific regulon member found in this study. The four conserved regulon members include an ATP-dependent RNA helicase-like protein (SrgF), two proteins likely involved in protein turnover (SrgKJ), truncated versions of a CDP-diacylglycerol pyrophosphatase and citrate transporter (SrgGH), and the menaquinone biosynthesis operon (*menFDHBCE*). The evolutionary maintenance of *sdiA-*dependent regulation at four loci suggests a common response to an AHL-laden environment.

Some *sdiA* regulon members have orthologs in other *sdiA*^+^ genera, but these have not been tested specifically for *sdiA*-dependent regulation in those genera. Therefore, we constructed transcriptional fusions to genes hypothesized to be regulated by *sdiA*, based on *sdiA*-dependent regulation in other genera. This led to three newly identified *sdiA* regulated loci in *E. cloacae* (citrate transporter *ENC_00800*, *srgKJ*, *menFDHBCE*), one in *E. coli* (*fepE*), and none in *Salmonella* (Fig. 4). This is the first reported instance of inter-genus conservation of the *sdiA* regulon. We have speculated on the existence of a “core regulon” common to all SdiA-mediated eavesdroppers that could link these apparently disparate responses to AHLs together. While we were successful in identifying loci conserved between two genera, no locus was identified that was conserved among all three.

Understanding the purpose of SdiA-mediated eavesdropping is hampered by the absence of *in vivo* and *in vitro* phenotypes. We hypothesized that one or both may be deduced from the *sdiA* regulon: applying known roles or functions of regulated genes to SdiA and elucidating environments from there. Although we were able to find new regulon members, there is very little known about them. SrgKJ, likely involved in protein turnover based on the activity of *E. coli* orthologs *qmcA-ybbJ*, has no reported defects or targets (85). The menaquinone biosynthesis operon (*menFDHBCE*) is known to be activated in anaerobic conditions, but we have no hypothesis as to its relationship to *sdiA*-mediated eavesdropping (87, 88). SrgF, a putative ATP-dependent RNA helicase-like protein, has not been characterized, but has been hit in several genetic screens (76–80). Those genetic screens suggested roles in colonization, motility, aquatic survival, and phage defense (76–80). We tested mutants lacking *srgF* or two other *sdiA* regulon members in serovar Typhimurium (*srgE*, *srgH*) for colonization defects in a mouse gastroenteritis model and observed no fitness defects (Fig. S2). Transcriptional fusions of *flhDC*, *fliA*, and *fliC* promoters had no *sdiA* or AHL-dependent differential expression in serovar Typhimurium (Fig. S1), and *sdiA* mutants of Typhimurium (47) and Typhi (data not shown) have no motility defects. Therefore, while *sdiA* is regulated by FliA, *sdiA* does not regulate motility in *Salmonella*. In *E. coli*, mutants of *yfgI* (orthologous to *srgL*) are reported to have a DNA repair defect. We tested both Typhi and Typhimurium for *sdiA-*dependent changes in sensitivity to DNA damage caused by either nalidixic acid or UV (Fig. S3) (91). No significant differences were found.

One explanation for the absence of colonization defects of *sdiA* and regulon mutants could be the lack of AHLs in the mouse gut. Our lab has previously found that SdiA is not active in the mouse gut and *sdiA* mutants have no fitness defect (17, 18). AHLs can be introduced into the gastrointestinal tract by co-infection with an AHL-producing pathogen (*Yersinia enterocolitica*) (17). Although *Salmonella* can detect those AHLs, *sdiA* mutants still have no fitness defects. An interesting effect is observed when wild-type and *sdiA* mutant *Salmonella* are co-infected in a genetic background encoding *yenI* from *Y. enterocolitica*, enabling *Salmonella* to produce AHLs without the need for another bacteria. The *sdiA* mutant is attenuated in the gut during the infection, and it is the largest observed phenotype of *sdiA* to our knowledge (>100-fold) (17). The differences in fitness phenotypes in an infection from foreign AHLs versus those endogenously produced may be due to *Yersinia*-specific factors (e.g., limited co-localization with *Salmonella,* anti-phagocytic activity) (111). Additional studies are needed to determine if AHLs are relevant to *Salmonella* in the gastrointestinal tract.

The study of SdiA-mediated eavesdropping has proven to be challenging. Numerous studies relating to *in vivo* and *in vitro* phenotypes have been performed with no clear answer as to the role of SdiA in the lifecycle of these bacteria. In terms of relevant environments, mammals and livestock are the most studied (17–19, 112, 113). Very few studies have been performed in insects (114) and plants (33, 115), which are colonized by both *sdiA*^+^ genera and AHL producers (including *Erwinia* and *Pantoea*) (116–123). Experiments in our lab indicate that serovar Typhimurium SdiA is active within house flies, but elucidating the fitness of the *sdiA* mutant requires additional study (A. Schwieters, B. M. M. Ahmer, unpublished data). We have also tested for *sdiA*-mediated gene regulation or fitness phenotypes in plants and rhizomes with no activity observed so far (A. Schwieters, B.M.M. Ahmer., unpublished).

Most *sdiA*-regulated genes are uncharacterized. We interpret this to mean that SdiA-mediated eavesdropping is part of a relatively unexplored aspect of these organisms’ lifestyle. There may be an interesting connection in *Enterobacter*, *E. coli,* and *Salmonella*: phage infection. Previous studies found *sdiA-*dependent regulation of a phage integrase in *E. cloacae* as well as prophage induction in *E. coli* (34, 60). In this study, we found several pieces of circumstantial evidence linking *Salmonella sdiA* to phage biology. First, expressing *sdiA* from a plasmid in serovar Typhimurium causes the downregulation of dozens of prophage genes (Table S4). Second, we found that *sdiA* represses transmission of the virulence plasmid pSLT, whose pilus is a likely phage target (124). We also found *sdiA-*dependent regulation of O-antigen chain length determinant *fepE* in both *E. coli* and *E. cloacae* (but not *Salmonella*)*,* which could potentially impact phage attachment. Finally, a transposon screen identified differential fitness of mutants during infection against certain phages: including *srgB*, *srgF*, *srgG,* and *sdiA* (80). Quorum sensing phage interactions have been previously reported in both directions [host regulation of phage defense and phage regulation of lysis-lysogeny decision making using quorum sensing receptors (106, 107, 125)]. Further study is needed to determine if *sdiA* plays a role in phage biology.

## MATERIALS AND METHODS

### Bacterial strains and media

Bacteria were grown in LB or on LB agar (1.5% wt/vol) unless otherwise stated. For motility experiments, agar was used at a final concentration of 0.25% wt/vol. Antibiotics were used at the following final concentrations: tetracycline (tet) at 10 µg/mL, kanamycin (kan) at 50 µg/mL, chloramphenicol (cam) at 30 µg/mL, ampicillin (amp) at 100 µg/mL, nalidixic acid (nal) at 50 µg/mL. Arabinose (ara) was used at a final concentration of 0.2%. N-(3-Oxooctanoyl)-DL-homoserine lactone (oxoC8) was obtained from Sigma Aldrich (Cat# O1639) and dissolved in ethyl acetate (EA) acidified with glacial acetic acid at a concentration of 0.1 mL/L (126). OxoC8 was used at a final concentration of 1 µM and acidified EA at 0.1% vol/vol. Ethylene glycol-bis(β-aminoethyl ether)-N,N,N′,N′-tetra-acetic acid (EGTA) was used at a final concentration of 10 mM. Anhydrotetracycline (AHT) was used at a final concentration of 5 µg/mL. Evans Blue Uranine (EBU) plates were made by adding tryptone (10 g/L), yeast extract (5 g/L), NaCl (5 g/L), glucose (2.5 g/L), and agar (15 g/L) to water, autoclaving, cooling to roughly 50°C, and then adding K_2_HPO_4_ (40 mL/L of 12.5% wt/vol), Evans Blue (1.25 mL/L of 1% wt/vol), and Uranine (also known as sodium fluorescein, 2.5 mL/L of 1% wt/vol) (127).

### Strain and plasmid construction

Strains and plasmids used in this study are listed in Table S1. Primers used in this study are listed in Table S2. New mutations were constructed as described below. Other strains were created by moving existing mutations into new strain backgrounds via P22 phage transduction. For P22 transductions, phage lysates were first grown on strains encoding the desired mutation. Recipient strains were then infected with the phage lysate for 25 minutes. The infection was halted by addition of LB + EGTA and outgrown for 1–3 hours before plating on selective media. Isolates were sub-cultured twice on selective media with EGTA, then cross-struck on EBU to confirm a lack of P22 pseudolysogeny and no P22 resistance mutations. The specific donor and recipients for each strain are described in Table S1. Plasmids were constructed as described below and moved into strains via electroporation (128).

### Chromosomal complementation strain AMS203

For chromosomal complementation, we inserted *sdiA* and its surrounding intergenic sequences between *pagC* and *STM14_1503,* which has been previously identified as a neutral insertion site (100). This position is depicted in Fig. 4D. Insertion of *sdiA* into the *pagC-STM14_1503* intergenic region was engineered by allelic exchange with suicide vector pFOK (129). The construct was assembled using Gibson assembly of four fragments: vector, the upstream region of *pagC* homology, the *sdiA* gene, and the downstream region of *STM14_1503* homology (130). The vector was linearized by PCR with primers BA3875 and BA3876. The upstream homology fragment was constructed by PCR with primers BA3883 and BA3884, which bind upstream of *STM14_1499* and downstream of *pagC*, respectively. The *sdiA* fragment was construct by PCR with primers BA3885 and BA3886, which bind immediately downstream of *yecC* and immediately downstream of *yecF*, respectively. The downstream homology fragment was constructed by PCR with primers BA3887 and BA3888, which bind downstream of *pagC* and within *pliC*, respectively. Primers include overhangs with homology to their adjacent fragments. PCR was performed with Q5 Polymerase; fragments were purified by gel extraction and quantified by Nanodrop. Gibson assembly was performed per manufacturer’s instruction.

Gibson product was transformed into TransforMax EC100D *pir*^+^ *E. coli* by electroporation (Lucigen ECP09500). The resulting plasmid, pAMS150, was moved into mating strain Jke201 by electroporation. Allelic exchange was performed by mating Jke201 + pAMS150 with BA612 on LB agar containing 100 µM diamino pimelic acid (DAP) then resuspending the bacteria and plating on LB kan to obtain single crossovers. Isolates were grown without selection and dilution plated on LB + AHT + 10% sucrose to select for a second crossover that eliminates the vector. Individual colonies were screened for loss of kan resistance and the insertion of *sdiA* was confirmed by PCR. The final strain is named AMS203.

### Construction of strains AMS001, AMS002, and JLD1221

Mutants of Typhi *sdiA* and Typhimurium *srgE* were created using Wanner mutagenesis (131). Chloramphenicol and kanamycin cassettes were amplified from pKD3 and pKD4, respectively. Primers BA3454 and BA3455 were used to generate insertions for mutants AMS001 and AMS002. Primers BA1563 and BA1564 were used to generate the insertion for JLD1221. Strains Ty2 and 14028 carrying helper plasmid pKD46 were transformed with gel purified DNA and isolated on selective media as previously described (131). The helper plasmid was eliminated from the strains by growth at 42°C. Mutations were confirmed by PCR. Strain AMS001 encodes the cam^r^ cassette oriented opposite *sdiA* while AMS002 encodes the kan^r^ cassette oriented with *sdiA*. Strain JLD1221 encodes the cam^r^ cassette oriented opposite *srgE*.

### Reporter plasmid construction

Transcriptional reporters of genes of interest were made by subcloning into luciferase reporter plasmid pSB401 (72). Promoters were amplified with Q5 Polymerase using primers listed in Table S2. Genomic DNA from strains 14028, Ty2, K12, and JLD401 served as the templates. DNA fragments were cloned into TOPO vector pCR2.1 (Invitrogen), and then removed by digestion with EcoRI and gel purified. The vector pSB401 was digested with EcoRI (NEB) and gel purified to remove the fragment encoding *luxR*. The vector and insert were ligated using T4 DNA ligase (NEB) and then transformed into chemically competent *E. coli*. Transformants were screened for insertion and orientation by PCR using forward primers binding the desired promoter and a universal reverse primer binding *luxC* downstream of the EcoRI site (BA1090). For transformation of plasmids into *Salmonella,* plasmids were first passaged through the restriction^-^ modification^+^ strain JS198.

The conditional expression plasmid pAMS130, encoding the *sdiA* gene from strain Ty2, was made by restriction cloning. The *sdiA* gene was amplified from the genome with primers BA3601 and BA3602. Vector pBAD33 was digested with SmaI. The digested vector and PCR product was blunt-end ligated using T4 DNA ligase, then transformed into competent cells (Stellar) and grown on selective media. Isolates were screened for insertion and orientation using two primer pairs: BA3601-BA2475 and BA3602-BA2474. Purified plasmid was transformed into strains by electroporation.

### RNA-seq and analysis

Overnight cultures of 14028, BA612, Ty2, and AMS001 were grown in LB at 37°C shaking. At a 1:100 dilution, they were sub-cultured in LB oxoC8 then incubated at 37°C shaking until late exponential phase. For plasmid over-expression, cultures of BA612 + pJVR2 and BA612 + pBAD33, AMS002 + pAMS130, and AMS002 + pBAD33 were grown overnight, supplemented with cam then sub-cultured in LB cam ara oxoC8. Three biological replicates were collected per strain. RNA was extracted from cell pellets using the PureLink RNA Mini Kit (Invitrogen #C12183018A) followed by DNase I treatment using TURBO DNA-free Kit (Invitrogen #AM2238). RNA quantity and quality was confirmed by Bioanalyzer. RNA was sent to the OSU Genomics Shared Resources Center for cDNA library synthesis and sequencing. Reads were assessed for quality and trimmed with FastQC and Trimmomatic, respectively (132, 133). Reads were mapped to *Salmonella* reference genomes (14028—accession number CP001363; Ty2—accession number AE014613) with Bowtie2 (134). Mapped reads were assembled, quantitated, and assigned to annotations using Stringtie (135). Differential expression analysis was performed using DESeq2 in R Studio (136). Results from the differential gene expression analysis provided log_2_ fold changes, *P*-values, and adjusted *P*-values for all genes. The adjusted *P*-value was calculated using the Benjamin-Hochberg method.

### Liquid and motility agar assays for lux reporter activity

Cultures of wild-type and *sdiA* mutants harboring reporter plasmids were grown shaking in LB with appropriate antibiotics at 37°C overnight. They were then sub-cultured 1:100 in LB broth or motility agar containing appropriate antibiotics and supplements (e.g. arabinose). For liquid assays, the bacteria were grown in a white plate with clear bottom, reading both OD_600_ and luminescence (Fisher Scientific, Cat# 265302). Measurements were taken every hour for 20 hours in the SpectraMax i3x at 37°C. Each sample was tested in technical triplicate per run, with three independent runs per strain or condition. For motility agar assays, only luminescence was measured. In both serovar Typhimurium and Typhi, no growth or motility defects were observed in *sdiA* mutants in either oxoC8 or a solvent control (data not shown).

### Conjugation assays

Conjugation assays for transmission of virulence plasmid pSLT were performed as previously described (97). Briefly, donor strains encode a plasmid marker, *spv::*Mu*d*J (kan^r^), and the recipient strain BA770 is a spontaneous nalidixic acid resistant mutant (nal^r^). Overnight cultures of donor and recipient were washed once in phosphate-buffered saline (PBS) and mated on LB agar (+ supplements) on a 0.45 µm filter disk at a multiplicity of infection (MOI) of 0.1. Disks were incubated overnight at 37°C. Filter disks were removed from the plate, resuspended in 3 mL PBS, and dilution plated on LB kan and LB kan nal to enumerate donors and transconjugants, respectively. Conjugation efficiency was calculated as the ratio of transconjugants to donors.

### DNA damage assays

To evaluate resistance to nalidixic acid, strains were grown overnight in LB at 37°C shaking. Cells were washed and diluted 1:100 into growth media (2 µL into 198 µL) in a 96-well plate. Endogenous expression strains were grown in LB + oxoC8 or LB + EA. Plasmid expression strains were grown in LB cam ± ara + oxoC8 or EA. Nalidixic acid was added into the media in a twofold dilution series from 50 to 0.15 µg/mL and a no nal control. The 50% inhibitory concentration (IC_50_) was calculated using GraphPad software (Prism Version 10) as the relative growth at 20 hours (OD_600_) compared to a no-antibiotic control and maximum concentration of antibiotic (no growth). Each strain and condition were tested on three separate occasions. For the disk diffusion assay, each strain was grown overnight in LB, washed, and inoculated into motility agar containing 1 µM oxoC8. Disks were inoculated with nalidixic acid in a twofold dilution series, starting at 250 µg (seven dilutions, one control) in a volume of 5 µL per disk. Plates were incubated overnight at 37°C, and images were taken in the morning. This was performed on three separate occasions.

To evaluate UV damage, strains were grown overnight in LB at 37°C shaking. Each strain was drip plated onto LB agar in a 10-fold dilution series. Once dry, plates were placed in a UV Crosslinker (HybriLinker HL-2000 UVP Laboratory Products). Plates were challenged with 0 to 150 × 100 µJ/CM^2^ in intervals of 25 µJ/CM^2^ (six conditions and one control). Plates were then grown overnight at 37°C and quantified. Survival was calculated as the ratio of colony forming units (CFU) at dose to CFU at no UV.

### Mouse experiments

All mice used in this study were 6- to 8-week-old female CBA/J mice purchased from Jackson Labs. This study used mice maintained on a high-fat diet, which confers susceptibility to inflammation and pathogen expansion in C57BL/6 (81) and CBA/J mice (A. Schwieters, B.M.M. Ahmer., unpublished data). The high-fat diet was purchased from vendor Research Diets Inc. (Cat#1705i) and provided 3 days prior to infection. Mice were maintained on the diet throughout the duration of the study. Wild-type and mutant strains were grown overnight in LB, washed in water, and mixed in a 1:1 ratio. Mice were orally gavaged to deliver 1 × 10^9^ CFU (day 0). Fecal pellets were collected and plated on LB containing selective media to quantify wild-type and mutant on days 1, 3, and 5. On day 7, mice were humanely euthanized, ceca were harvested and plated for CFU on selective media. The fitness of *Salmonella* mutants was compared to that of the wild-type by calculating the ratio of the mutant to the wild-type divided by initial ratio of mutant to wild-type (~1:1). Values below 1 [or negative log_10_(CI)] indicate fitness defects in the mutant.

## Data Availability

The RNA-seq data have been deposited in the NCBI Gene Expression Omnibus repository under accession number GSE275322.

## SUPPLEMENTAL MATERIAL

Table S1 spectrum.01929-24-s0001.docx Strains and plasmids used in study.

Fig. S1 spectrum.01929-24-s0002.tif Reporter plasmids responsive to plasmid SdiA but not chromosomal.

Fig. S2 spectrum.01929-24-s0003.tif Reporter plasmids not activated by plasmid SdiA or chromosomal SdiA.

Fig. S3 spectrum.01929-24-s0004.tif Phenotypes of SdiA regulated genes.

Supplemental material spectrum.01929-24-s0005.pdf Legends for Fig. S1 to S3.

Supplemental tables spectrum.01929-24-s0006.xlsx Tables S3 to S6.

Table S2 spectrum.01929-24-s0007.docx Primers used in study.

ASM does not own the copyrights to Supplemental Material that may be linked to, or accessed through, an article. The authors have granted ASM a non-exclusive, world-wide license to publish the Supplemental Material files. Please contact the corresponding author directly for reuse.
