# Supplementary material for: Identification of new SdiA regulon members of Escherichia coli, Enterobacter cloacae, and Salmonella enterica serovars Typhimurium and Typhi
Source: Microbiol Spectr. 2024 Oct 22;12(12):e01929-24. doi: 10.1128/spectrum.01929-24 (PMC11619404; doi:10.1128/spectrum.01929-24)
Supplement: Table S1 — Strains and plasmids used in study. [file spectrum.01929-24-s0001.docx]

**Table S1: List of strains and plasmids used in this study.**

| **Strain** | **Genotype or Description** | **Source, Construction, or Reference** |
| --- | --- | --- |
| ATCC 14028 (14028) | Wild-type *Salmonella* *enterica* subspecies  *enterica* serovar Typhimurium strain 14028 | American Type Culture Collection (ATCC) |
| BA612 | 14028 *sdiA*::mTn3 | [1] |
| JSG624 (Ty2) | Wild-type *Salmonella enterica* subspecies *enterica* serovar Typhi strain Ty2 | John Gunn |
| AMS001 | Ty2 *sdiA1*::cam. Made by Wanner mutagenesis with primers BA3454 and BA3455 | This Study |
| AMS002 | Ty2 *sdiA2*::kan. Made by Wanner mutagenesis with primers BA3454 and BA3455 | This Study |
| AMS203 | BA612 *pagC* IG::*sdiA1* | This Study |
| AMS246 | AMS203 *spv154::*MudJ. P22 transduction P22_BA1541_ X AMS203 | This Study |
| AMS171 | BA612 *spv1541*::MudJ. P22 transduction P22_BA1541_ X BA612 | This Study |
| BA1541 | 14028 *spv1541*::MudJ | [2] |
| BA770 | 14028 – pSLT nal^R^ | [2] |
| Jke201 | Mating strain of *E. coli*, see reference [3] for full genotype and description | Gift from Dirk Bumann, [3] |
| JLD401 | *Enterobacter cloacae* Nal^R^ | [4] |
| ASD401 | JLD401 *sdiA32*::mTn5-FC | [4] |
| MG1655 | Wild-type Escherichia coli K-12 strain MG1655 | *E. coli* Genetic Stock Center |
| JNS21 | MG1655 *sdiA25*::EZ-Tn5,kan-2. | [5] |
| JS198 | LT2 *metE551* *metA22* *ilv452* *trpB2* *hisC527(am) galE496 xyl-404 rpsL120*  *flaA66 hsdL6 hsdSA29 zjg8103 : :* pir+ *recA1* | [6] |
| AMS254 | 14028 *srgF1::*kan.  P22 transduction P22*_srgF1::_*_kan_ X 14028 | This Study |
| AMS264 | 14028 *srgH1::*kan.  P22 transduction P22*_srgH1::_*_kan_ X 14028 | This Study |
| JLD1214 | 14028 *IG(pagC-STM14_1502)*::Cam | [7] |
| EFB051 | 14028 *IG(pagC-STM14_1502)6*::Kan | [8] |
| JLD1221 | 14028 *srgE42::cam*. Constructed by wanner mutagenesis. Insertion amplified with primers BA1563 and BA1564. Transduced into a clean 14028 background by P22 | This Study |
| *srgF1::*kan | Mutation from McClellend Collection | [9] |
| *srgH1::*cam | Mutation from McClellend Collection | [9] |
| **Plasmid** | **Genotype or Description** | **Source, Construction, or Reference** |
| pKD46 | PBAD *gam* *bet* *exo* pSC101 oriTS | [10] |
| pKD3 | FRT-cam-FRT *oriR6K* | [10] |
| pKD4 | FRT-kan-FRT *oriR6K* | [10] |
| pBAD33 | pACYC vector for arabinose-conditional expression | [11] |
| pAMS130 | pBAD33 *sdiA^+^* (Typhi). Insert amplified with primers BA3601 and BA3602 | This Study |
| pJVR2 | pBAD33 *sdiA^+^* (Typhimurium) | [1] |
| pBAD18 | ColE1 origin vector for arabinose-conditional expression | [11] |
| pSB401 | *luxR^+^ luxI::luxCDABE* | [12] |
| pBA321 | pBAD18 *sdiA^+^* (Typhimurium) | [13] |
| pFOK | Suicide vector for allelic exchange. | Dirk Bumann [14] |
| pAMS150 | pFOK-SdiA. See methods | This Study |
| pAMS148 | Reporter Plasmid of Typhimurium *srgF* (*STM14_3820*). Insert amplified with primers BA3869 and BA3870. | This Study |
| pAMS145 | Reporter Plasmid of Typhimurium *srgGH* (*STM14_4893-4894*). Insert amplified with primers BA3863 and BA3864 | This Study |
| pJLD202 | Reporter Plasmid of Typhimurium *srgKJ* (*STM14_0589-0588*). Insert amplified with primers BA1218 and BA1219. | [15] disseration |
| pAMS291 | Reporter Plasmid of Typhimurium *menFDHBCE* (*STM14_2848-2843*). Insert amplified with primers BA4031 and BA4032 | This Study |
| pJNS25 | Reporter Plasmid of Typhimurium *srgE* (*STM14_1877*). | [16] |
| pBA428 | Reporter Plasmid of Typhimurium *pefI-srgC*. | [13] |
| pAMS205 | Reporter Plasmid of Typhi *srgF* (*T06040*). Insert amplified with primers BA3953 and BA3964 | This Study |
| pAMS265 | Reporter Plasmid of Typhi *srgGH* (*T3549-3548*). Insert amplified with primers BA4025 and BA4026 | This Study |
| pAMS050 | Reporter Plasmid of Typhi *srgKJ* (*T2359-2360*). Insert amplified with primers BA3710 and BA3711 | This Study |
| pAMS202 | Reporter Plasmid of Typhi *menFDHBCE* (*T0553-0558*). Insert amplified with primers BA3950 and BA3961 | This Study |
| pAMS048 | Reporter Plasmid of Typhi *srgE* (*T1468*). Insert amplified with primers BA3706 and BA3707 | This Study |
| pAMS347 | Reporter Plasmid of Typhi *srgDAB* (*T4538-4540*). Insert amplified with primers BA4075 and BA4076 | This Study |
| pAMS201 | Reporter Plasmid of Typhi (*T0351-0350*). Insert amplified with primers BA3949 and BA3960 | This Study |
| pAMS042 | Reporter Plasmid of Typhimurium *yjiXA* (*STM14_5444-5443*). Insert amplified with primers BA3722 and BA3723 | This Study |
| pAMS055 | Reporter Plasmid of Typhimurium *yjiA* (*STM14_5443*). Insert amplified with primers BA3720 and BA3721 | This Study |
| pAMS096 | Reporter Plasmid of Typhimurium *yjiYXA* (*STM14_5445-5443*). Insert amplified with primers BA3828 and BA3829 | This Study |
| pAMS043 | Reporter Plasmid of Typhimurium *ybdNM* (*STM14_0704-0703*). Insert amplified with primers BA3724 and BA3725 | This Study |
| pAMS097 | Reporter Plasmid of Typhimurium *ybdO* (*STM14_0705*). Insert amplified with primers BA3830 and BA3831 | This Study |
| pRG38 | Reporter Plasmid of Typhimurium *flhD* (*STM14_2341*). | [17] |
| pRG34 | Reporter Plasmid of Typhimurium *fliA* (*STM14_2374*). | [17] |
| pRG39 | Reporter Plasmid of Typhimurium *fliC* (*STM14_2378*). | [17] |
| pDL05 | Reporter Plasmid of Typhimurium *rtsA* (*STM14_5188*). Insert amplified with primers BA1631 and BA1632 | [18] |
| pDL83 | Reporter Plasmid of Typhimurium *invF* (*STM14_3498*). Insert amplified with primers BA1978 and BA1979 | [18] |
| pBA409 | Reporter Plasmid of Typhimurium *sopB* (*STM14_1237*). | [17] |
| pAMS144 | Reporter Plasmid of Typhimurium *yecF* (*STM14_2367*). Insert amplified with primers BA3861 and BA3862 | This Study |
| pAMS146 | Reporter Plasmid of Typhimurium *yciG* (*STM14_2091*). Insert amplified with primers BA3865 and BA3866 | This Study |
| pAMS147 | Reporter Plasmid of Typhimurium *STM14_1829*. Insert amplified with primers BA3867 and BA3868 | This Study |
| pAMS188 | Reporter Plasmid of Typhimurium *ybdM* (*STM14_0703*). Insert amplified with primers BA3946 and BA3948 | This Study |
| pAMS187 | Reporter Plasmid of Typhimurium *pdxJ-acpS* (*STM14_3158-3157*). Insert amplified with primers BA3945 and BA3947 | This Study |
| pAMS143 | Reporter Plasmid of Typhimurium *dpiA* (*STM14_0728*). Insert amplified with primers BA3859 and BA3860 | This Study |
| pAMS184 | Reporter Plasmid of Typhimurium *citA* (*STM14_0804*). Insert amplified with primers BA3860 and BA3859 | This Study |
| pMT45 | Reporter Plasmid of Typhimurium *fimA* (*STM14_0635*). | [19] |
| pAMS154 | Reporter Plasmid of Typhimurium *STM14_0979*. Insert amplified with primers BA3889 and BA3890 | This Study |
| pAMS156 | Reporter Plasmid of Typhimurium *ynfL* (*STM14_1798*). Insert amplified with primers BA3893 and BA3894 | This Study |
| pAMS172 | Reporter Plasmid of Typhimurium *proVWX* (*STM14_3391-3393*). Insert amplified with primers BA3902 and BA3914 | This Study |
| pAMS178 | Reporter Plasmid of Typhimurium *rnc-acpS* (*STM14_3161-3157*). Insert amplified with primers BA3908 and BA3920 | This Study |
| pAMS179 | Reporter Plasmid of Typhimurium *acpS* (*STM14_3157*). Insert amplified with primers BA3909 and BA3921 | This Study |
| pMT47 | Reporter Plasmid of Typhimurium *fimY* (*STM14_0642*). | [19] |
| pMT48 | Reporter Plasmid of Typhimurium *fimW* (*STM14_0644*). | [19] |
| pAMS173 | Reporter Plasmid of Typhimurium *leuABCD* (*STM14_0134-0131*). Insert amplified with primers BA3903 and BA3915 | This Study |
| pAMS174 | Reporter Plasmid of Typhimurium *btuCED* (*STM14_1627-1629*). Insert amplified with primers BA3906 and BA3918 | This Study |
| pAMS175 | Reporter Plasmid of Typhimurium *btuED* (*STM14_1628-1629*). Insert amplified with primers BA3905 and BA3917 | This Study |
| pAMS362 | Reporter Plasmid of *E. cloacae* *menFDHBCE*. Insert amplified with primers BA4081 and BA4082 | This Study |
| pAMS228 | Reporter Plasmid of *E. cloacae* *ybbKJ*. Insert amplified with primers BA4003 and BA4004 | This Study |
| pAMS360 | Reporter Plasmid of *E. cloacae* *srgF* (*ENC_32410*). Insert amplified with primers BA4077 and BA4078 | This Study |
| pAMS368 | Reporter Plasmid of *E. cloacae* *ENC_00800*. Insert amplified with primers BA4093 and BA4094 | This Study |
| pAMS367 | Reporter Plasmid of Typhimurium *fepE* (*STM14_0687*). Insert amplified with primers BA4091 and BA4092 | This Study |
| pAMS231 | Reporter Plasmid of Typhimurium *copA* (*STM14_0586*). Insert amplified with primers BA4001 and BA4002 | This Study |
| pAMS364 | Reporter Plasmid of E. coli *menFDHBCE*. Insert amplified with primers BA4085 and BA4086 | This Study |
| pAMS227 | Reporter Plasmid of *E. coli* *ybbKJ*. Insert amplified with primers BA3999 and BA4000 | This Study |
| pAMS363 | Reporter Plasmid of *E. coli* *yfgHI*. Insert amplified with primers BA4083 and BA4084 | This Study |
| pAMS361 | Reporter Plasmid of *E. cloacae* *fliE*. Insert amplified with primers BA4079 and BA4080 | This Study |
| pAMS366 | Reporter Plasmid of *E. coli* *fepE*. Insert amplified with primers BA4089 and BA4090 | This Study |

1. Ahmer, B.M.M., et al., *Salmonella typhimurium Encodes an SdiA Homolog, a Putative Quorum Sensor of the LuxR Family, That Regulates Genes on the Virulence Plasmid.* Journal of Bacteriology, 1998. **180**(5): p. 1185-1193.

2. Ahmer, B.M.M., M. Tran, and F. Heffron, *The Virulence Plasmid of Salmonella typhimurium Is Self-Transmissible.* Journal of Bacteriology, 1999. **181**(4): p. 1364-1368.

3. Harms, A., et al., *A bacterial toxin-antitoxin module is the origin of inter-bacterial and inter-kingdom effectors of Bartonella.* PLOS Genetics, 2017. **13**(10): p. e1007077.

4. Sabag-Daigle, A., et al., *Identification of sdiA-regulated genes in a mouse commensal strain of Enterobacter cloacae.* Frontiers in Cellular and Infection Microbiology, 2015. **5**(47).

5. Dyszel, J.L., et al., *E. coli K-12 and EHEC Genes Regulated by SdiA.* PLOS ONE, 2010. **5**(1): p. e8946.

6. Ellermeier, C.D., A. Janakiraman, and J.M. Slauch, *Construction of targeted single copy lac fusions using lambda Red and FLP-mediated site-specific recombination in bacteria.* Gene, 2002. **290**(1-2): p. 153-61.

7. Ali, M.M., et al., *Fructose-Asparagine Is a Primary Nutrient during Growth of Salmonella in the Inflamed Intestine.* PLOS Pathogens, 2014. **10**(6): p. e1004209.

8. Boulanger, E.F., et al., *Sugar-Phosphate Toxicities Attenuate Salmonella Fitness in the Gut.* Journal of Bacteriology, 2022. **0**(0): p. e00344-22.

9. Santiviago, C.A., et al., *Analysis of Pools of Targeted Salmonella Deletion Mutants Identifies Novel Genes Affecting Fitness during Competitive Infection in Mice.* PLOS Pathogens, 2009. **5**(7): p. e1000477.

10. Datsenko, K.A. and B.L. Wanner, *One-step inactivation of chromosomal genes in Escherichia coli K-12 using PCR products.* Proceedings of the National Academy of Sciences, 2000. **97**(12): p. 6640-6645.

11. Guzman, L.M., et al., *Tight regulation, modulation, and high-level expression by vectors containing the arabinose PBAD promoter.* Journal of Bacteriology, 1995. **177**(14): p. 4121-4130.

12. Winson, M.K., et al., *Construction and analysis of luxCDABE-based plasmid sensors for investigating N-acyl homoserine lactone-mediated quorum sensing.* FEMS Microbiology Letters, 1998. **163**(2): p. 185-192.

13. Michael, B., et al., *SdiA of Salmonella enterica Is a LuxR Homolog That Detects Mixed Microbial Communities.* Journal of Bacteriology, 2001. **183**(19): p. 5733-5742.

14. Cianfanelli, F.R., O. Cunrath, and D. Bumann, *Efficient dual-negative selection for bacterial genome editing.* BMC Microbiology, 2020. **20**(1): p. 129.

15. Dyszel, J.L., *Phenotypes of the LuxR homolog, SdiA, in Salmonella and Escherichia coli*. 2009, The Ohio State University: United States -- Ohio. p. 171.

16. Smith, J.N. and B.M.M. Ahmer, *Detection of Other Microbial Species by Salmonella: Expression of the SdiA Regulon.* Journal of Bacteriology, 2003. **185**(4): p. 1357-1366.

17. Goodier, R.I. and B.M.M. Ahmer, *SirA Orthologs Affect both Motility and Virulence.* Journal of Bacteriology, 2001. **183**(7): p. 2249-2258.

18. Lucas, D.E., *Coordinated Regulation of Salmonella Virulence Genes by the BarA/SirA Two-Component System and the Csr Global Regulatory System*. 2013, The Ohio State University: United States -- Ohio. p. 219.

19. Teplitski, M., A. Al-Agely, and B.M.M. Ahmer, *Contribution of the SirA regulon to biofilm formation in Salmonella enterica serovar Typhimurium.* Microbiology, 2006. **152**(11): p. 3411-3424.
