# Supplementary figures and images for: Identification of new SdiA regulon members of Escherichia coli, Enterobacter cloacae, and Salmonella enterica serovars Typhimurium and Typhi
Source: Microbiol Spectr. 2024 Oct 22;12(12):e01929-24. doi: 10.1128/spectrum.01929-24 (PMC11619404; doi:10.1128/spectrum.01929-24)

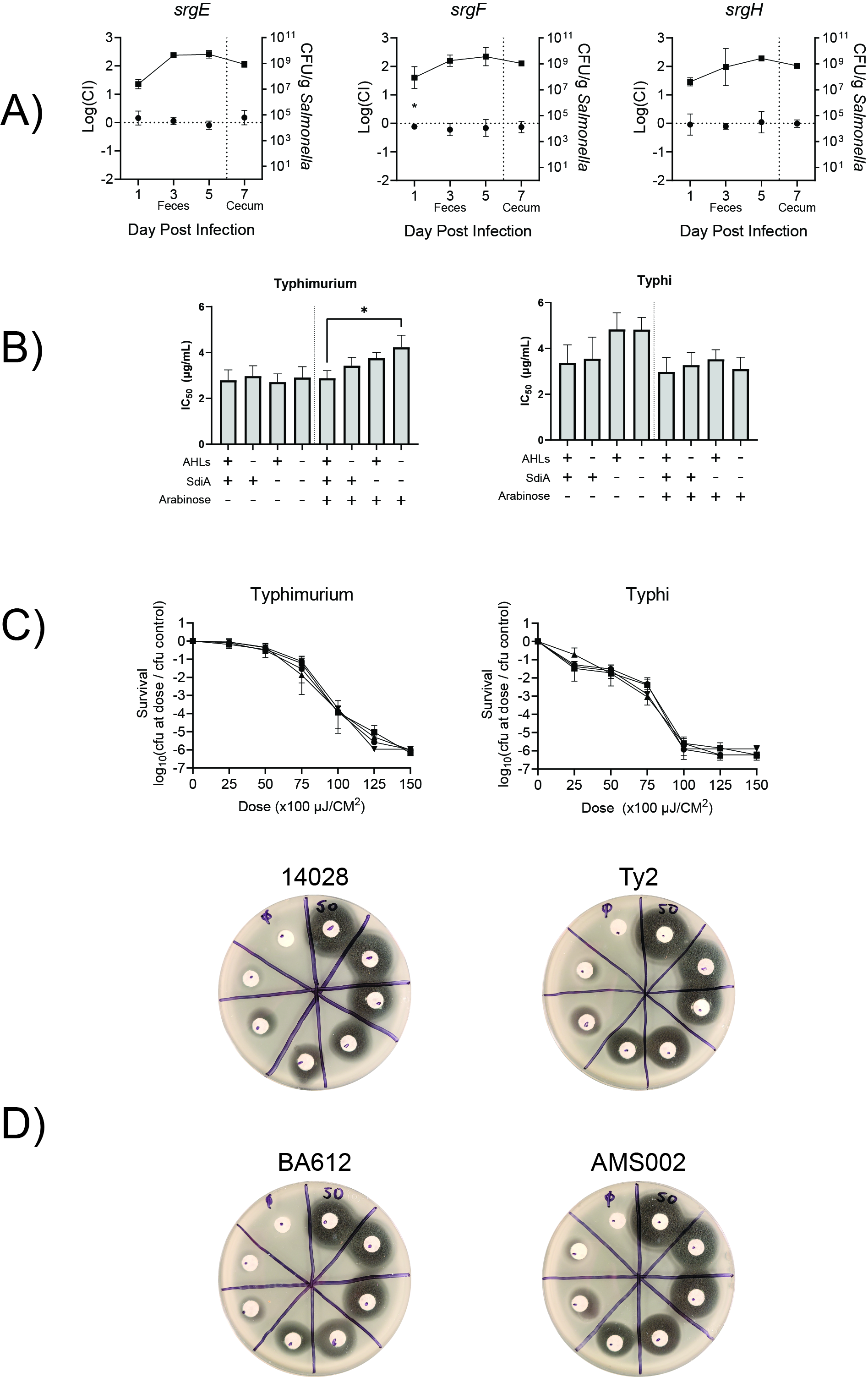

Supplement: Fig. S3 — Phenotypes of SdiA regulated genes. [file spectrum.01929-24-s0004.tif]
