# Supplementary material for: Identification of new SdiA regulon members of Escherichia coli, Enterobacter cloacae, and Salmonella enterica serovars Typhimurium and Typhi
Source: Microbiol Spectr. 2024 Oct 22;12(12):e01929-24. doi: 10.1128/spectrum.01929-24 (PMC11619404; doi:10.1128/spectrum.01929-24)
Supplement: Supplemental material — Legends for Fig. S1 to S3. [file spectrum.01929-24-s0005.pdf]

Figure S1. Reporters that respond to plasmid-based expression of *sdiA*, but not *sdiA* expressed from its natural position in the chromosome.

Diagrams of reporter fusion constructs and their expression data for putative *sdiA*-regulated genes in serovar Typhimurium. This figure includes reporter constructs that are regulated by *sdiA* expressed from a plasmid but not from the chromosome. Diagrams show the genomic context of each gene of interest identified by RNA-seq (blue) and the putative promoter region cloned into pSB401 to measure transcriptional activity (orange arrow). Size of each figure is not to scale with length of region represented. Each reporter was tested for *sdiA*-dependent regulation in strain backgrounds expressing sdiA from a plasmid or from the chromosome in motility agar or LB. In motility agar graphs, Y-axes represent raw light units. In LB graphs, Y-axes represent raw light units normalized to growth (OD_{600}) at the corresponding time point. Each time point is mean +/- SD of 9 replicates (3 technical x 3 biological).

For *sdiA* expressed from a plasmid, the strains are: *sdiA*$^+$ – BA612 + pBA321, *sdiA* mutant – BA612 + pBAD18. For sdiA expressed from the chromosome, the strains are: *sdiA*$^+$ – 14028, *sdiA* mutant - BA612

Open circles: *sdiA*$^+$ + solvent (EA). Closed circles: *sdiA*$^+$ + AHL. Open squares: *sdiA* mutant + solvent (EA). Closed squared: *sdiA* mutant + AHL. When using *sdiA* expressed from a plasmid, all media were supplemented with arabinose (0.2%).

Figure S2. Reporters that do not respond to plasmid-based expression of *sdiA*, or *sdiA* expressed from its natural position in the chromosome.

Diagrams of reporter fusion constructs and their expression data for putative *sdiA*-regulated genes in serovar Typhimurium. This figure includes reporter constructs that are not regulated by *sdiA* under any condition tested to date. Diagrams show the genomic context of each gene of interest identified by RNA-seq (blue) and the putative promoter region cloned into pSB401 to measure transcriptional activity (orange arrow). Size of each figure is not to scale with length of region represented. Each reporter was tested for *sdiA*-dependent regulation in strain backgrounds expressing sdiA from a plasmid or from the chromosome in motility agar or LB. In motility agar graphs, Y-axes represent raw light units. In LB graphs, Y-axes represent raw light units normalized to growth (OD_{600}) at the corresponding time point. Each time point is mean +/- SD of 9 replicates (3 technical x 3 biological).

For *sdiA* expressed from a plasmid, the strains are: *sdiA*$^+$ – BA612 + pBA321, *sdiA* mutant – BA612 + pBAD18. For sdiA expressed from the chromosome, the strains are: *sdiA*$^+$ – 14028, *sdiA* mutant - BA612

Open circles: *sdiA*$^+$ + solvent (EA). Closed circles: *sdiA*$^+$ + AHL. Open squares: *sdiA* mutant + solvent (EA). Closed squared: *sdiA* mutant + AHL. When using *sdiA* expressed from a plasmid, all media were supplemented with arabinose (0.2%).

Figure S3. Tested phenotypes of SdiA and regulated genes.

A) Competition assays between wild-type (14028) and mutants of *sdiA*-regulated genes. SrgE competition (EFB051 vs JLD1221), SrgF competition (JLD1214 vs AMS254), SrgH (EFB051 vs

AMS264). Mice were inoculated orally with both strains in a 1:1 ratio. Fecal pellets were collected and CFU quantified (squares, right axis) and competitive index (circles, left axis). Cecum was collected and CFU quantified on Day 7. Competitive index at each time point was calculated as the ratio of mutant to wild-type divided by the ratio of mutant to wild-type in the inoculum. Each competition was performed with five female CBA/J mice. Statistical significance was evaluated using a one-sample student's t-test. * $P < 0.05$

B) Minimum inhibitory concentration of nalidixic acid for serovars Typhimurium and Typhi. See methods for details on growth conditions and IC_{50} calculations. Strains used in assay: Typhimurium – 14028, BA612, BA612 + pJVR2, BA612 + pBAD18; Typhi – Ty2, AMS002, AMS002 +pAMS130, AMS002 + pBAD33. The left four strains utilize endogenous expression of *sdiA*. The right four strains utilize sdiA expressed from a plasmid. Mean and standard deviation was calculated from three independent experiments. Significance was evaluated using a student's t-test. * $P < 0.05$

C) Resistance of serovars Typhimurium and Typhi to UV-mediated killing. See methods for details on assay. Circles – 14028 and Ty2, squares – BA612 and AMS002, upward triangle BA612 + pJVR2 and AMS002 + pAMS130, downward triangle BA612 + pBAD33 and AMS002 + pBAD33. No timepoints were significant as determined by student's t tests. Mean and standard deviation was calculated from three independent experiments.

D) Resistance of serovars Typhimurium and Typhi to nalidixic acid in motility agar. Strains were inoculated into motility agar containing 1 μM AHL. Disks were implanted with indicated quantities of Nalidixic Acid in 5 μL of water. Plates were incubated overnight at 37 °C. Images are representative of three independent experiments.
