# Supplementary material for: Identification of new SdiA regulon members of Escherichia coli, Enterobacter cloacae, and Salmonella enterica serovars Typhimurium and Typhi
Source: Microbiol Spectr. 2024 Oct 22;12(12):e01929-24. doi: 10.1128/spectrum.01929-24 (PMC11619404; doi:10.1128/spectrum.01929-24)
Supplement: Table S2 — Primers used in study. [file spectrum.01929-24-s0007.docx]

**Table S2: List of primers used in this study.**

| **Primer** | **Sequence** | **Description** |
| --- | --- | --- |
| BA1090 | GAATGTATGTCCTGCGTCTTGAGTA | Universal reverse verification primer for pSB401 reporter constructs |
| BA1218 | AGGGCTTATTAACGAGGCCACCATT | Primer for amplification of insert in reporter plasmid pJLD202 |
| BA1219 | TTGGTCATGGTCAGGTTAATGATCG | Primer for amplification of insert in reporter plasmid pJLD202 |
| BA1563 | AGTGAAGCTATACCTAACGTGGCTGTTCCTGCAAAATGTGTAGGCTGGAGCTGCTTCG | Primer for generating mutant JLD1221 |
| BA1564 | TAGATTCATCCTGAAAGAGCTAATTAGCTCTCCCGACATATGAATATCCTCCTTAG | Primer for generating mutant JLD1221 |
| BA1631 | TACCTCATGCTAACTACCTCC | Primer for amplification of insert in reporter plasmid pDL05 |
| BA1632 | TGGGGCCGAAAAGTCTGCATGTT | Primer for amplification of insert in reporter plasmid pDL05 |
| BA1978 | GAAGAAGGTGAGCGCCTGTTCTTTG | Primer for amplification of insert in reporter plasmid pDL83 |
| BA1979 | CGATCTTGCCAAATAGCGCGAAACTC | Primer for amplification of insert in reporter plasmid pDL83 |
| BA2474 | ACCACCCCCTGACCGCGAATGGTGA | Verification primer for insertions into pBAD18 and pBAD33 vectors |
| BA2475 | AAGCATTTATCAGGGTTATTGTCTC | Verification primer for insertions into pBAD18 and pBAD33 vectors |
| BA3454 | GACCATAAAATATGCAGGAAAATGATTTCTTCACCTGGCGGTGTAGGCTGGAGCTGCTTC | Primer for generating mutants AMS001 and AMS002 |
| BA3455 | CGTCAGCACGTCATATCAGACCTGTCGCCGCAGCGTAGCACATATGAATATCCTCCTTAG | Primer for generating mutants AMS001 and AMS002 |
| BA3601 | ATGCAGGAAAATGATTTCTT | Primer for amplifying Typhi *sdiA* in construction of pAMS130 |
| BA3602 | TCATATCAGACCTGTCGCCG | Primer for amplifying Typhi *sdiA* in construction of pAMS130 |
| BA3706 | GACATCATAAGCTTCACATAATAAAA | Primer for amplification of insert in reporter plasmid pAMS048 |
| BA3707 | ATAAGTAGCGTAATCCATTTTTCTAT | Primer for amplification of insert in reporter plasmid pAMS048 |
| BA3710 | CTGTAGATTACGAATTAGAGCAATAC | Primer for amplification of insert in reporter plasmid pAMS050 |
| BA3711 | AATTTCGATACGGGTAACTTTAATTC | Primer for amplification of insert in reporter plasmid pAMS050 |
| BA3720 | ATCATTGAACCGGGTCTCTACTTC | Primer for amplification of insert in reporter plasmid pAMS055 |
| BA3721 | CATAATACGTCATGGGAGAAAAAG | Primer for amplification of insert in reporter plasmid pAMS055 |
| BA3722 | CTTTGTGATTTCCCCGGAACAAATC | Primer for amplification of insert in reporter plasmid pAMS042 |
| BA3723 | TAGTCCGGAATACCAATCAACATTTT | Primer for amplification of insert in reporter plasmid pAMS042 |
| BA3724 | AATAATCGTTCTATTGTTTGTACTCA | Primer for amplification of insert in reporter plasmid pAMS043 |
| BA3725 | TGATACATCAAATTATACAAAGGGTT | Primer for amplification of insert in reporter plasmid pAMS043 |
| BA3828 | TGGTAGATATCATAGGTTCGTTTGAT | Primer for amplification of insert in reporter plasmid pAMS096 |
| BA3829 | CGAGGATGATGATCATGATTAAGAAG | Primer for amplification of insert in reporter plasmid pAMS096 |
| BA3830 | TCATTAATCAGAATCAGCAGCTAATG | Primer for amplification of insert in reporter plasmid pAMS097 |
| BA3831 | AATTCCTTTGCCTGAACGAATAAATA | Primer for amplification of insert in reporter plasmid pAMS097 |
| BA3859 | CATCACAATACAGCCAATTTTCTTTC | Primer for amplification of insert in reporter plasmid pAMS143/pAMS184 |
| BA3860 | ATTTTACTGACCAGATAGCCAATTGA | Primer for amplification of insert in reporter plasmid pAMS143/pAMS184 |
| BA3861 | CATGATCACTTTGATATCCGCTGTC | Primer for amplification of insert in reporter plasmid pAMS144 |
| BA3862 | TACCATAAGCTACGCTAAAAATAGCA | Primer for amplification of insert in reporter plasmid pAMS144 |
| BA3863 | ACTATCTCTATATTTCGCGTATTCGT | Primer for amplification of insert in reporter plasmid pAMS145 |
| BA3864 | AAAAATAGCAGTGCGGTCATAAACTC | Primer for amplification of insert in reporter plasmid pAMS145 |
| BA3865 | TTGTGAACAGGTTGGCGTAGATTC | Primer for amplification of insert in reporter plasmid pAMS146 |
| BA3866 | GTTGCGGATCGTTTTTGAAATTC | Primer for amplification of insert in reporter plasmid pAMS146 |
| BA3867 | GGTGGCTTGATTGCCAAAGATTTATT | Primer for amplification of insert in reporter plasmid pAMS147 |
| BA3868 | CTTCTGAGGCTTTCTCTTTATCTTCT | Primer for amplification of insert in reporter plasmid pAMS147 |
| BA3869 | GTTTCAATTTTAGCCACACAATACAG | Primer for amplification of insert in reporter plasmid pAMS148 |
| BA3870 | ATTAATACTGAGAAATGATCTTCGCC | Primer for amplification of insert in reporter plasmid pAMS148 |
| BA3875 | ATCGAATTCCTGCAGCCCGGGGGATCCACT | Primer for construction of suicide vector pAMS150. See methods |
| BA3876 | ATCAAGCTTATCGATACCGTCGACCTCGAG | Primer for construction of suicide vector pAMS150. See methods |
| BA3883 | CTCGAGGTCGACGGTATCGATAAGCTTGATATCGGAGCGGGAATAAAGCG | Primer for construction of suicide vector pAMS150. See methods |
| BA3884 | AGCGTTGAAAAGGCAGAGAGAAAAGACAGGCAGGT | Primer for construction of suicide vector pAMS150. See methods |
| BA3885 | CCTGTCTTTTCTCTCTGCCTTTTCAACGCTCGC | Primer for construction of suicide vector pAMS150. See methods |
| BA3886 | ACATTGTGATTAATTTAAAAAACCGGCTGTTAGCATCG | Primer for construction of suicide vector pAMS150. See methods |
| BA3887 | ACAGCCGGTTTTTTAAATTAATCACAATGTCATCAAGA | Primer for construction of suicide vector pAMS150. See methods |
| BA3888 | AGTGGATCCCCCGGGCTGCAGGAATTCGATAAATTACGAAGCCATAGACA | Primer for construction of suicide vector pAMS150. See methods |
| BA3889 | TTTTTATTTTTTCCGAATGCAATGTG | Primer for amplification of insert in reporter plasmid pAMS154 |
| BA3890 | ACAATATGTTTACCACAAAATATATTCG | Primer for amplification of insert in reporter plasmid pAMS154 |
| BA3893 | AATAGCTGAAAAGATAAAGTGACGAG | Primer for amplification of insert in reporter plasmid pAMS156 |
| BA3894 | GTATTCATTTCACGCGTTTGCATAT | Primer for amplification of insert in reporter plasmid pAMS156 |
| BA3902 | AGATTATTTTAGCTCATTACGTCAGC | Primer for amplification of insert in reporter plasmid pAMS172 |
| BA3903 | TCTACACGATTATAAATCTGTGACGT | Primer for amplification of insert in reporter plasmid pAMS173 |
| BA3905 | TAATGTACTGGATGATGGGAGGATTT | Primer for amplification of insert in reporter plasmid pAMS175 |
| BA3906 | CGTCTTTATTGAGCATAACGATAACT | Primer for amplification of insert in reporter plasmid pAMS174 |
| BA3908 | CGAGCGATTTTGTACAGACTTTT | Primer for amplification of insert in reporter plasmid pAMS178 |
| BA3909 | ATCCAGGTTTCGCTCTTTATCGAT | Primer for amplification of insert in reporter plasmid pAMS179 |
| BA3914 | GGAAAACGCTTCATCCATTAATAAGA | Primer for amplification of insert in reporter plasmid pAMS172 |
| BA3915 | CTGGCGTCATGATTTCATAGTTTT | Primer for amplification of insert in reporter plasmid pAMS173 |
| BA3917 | CACAATAGGTTTTAATCTCCTCTTCG | Primer for amplification of insert in reporter plasmid pAMS175 |
| BA3918 | GTTAGAGGTACACAGCACGTTAC | Primer for amplification of insert in reporter plasmid pAMS174 |
| BA3920 | GGAATCCGCCGCTTTTTAATTC | Primer for amplification of insert in reporter plasmid pAMS178 |
| BA3921 | ATCGTTAAACACTTCGAACTGATTG | Primer for amplification of insert in reporter plasmid pAMS179 |
| BA3945 | TCATCTGGGGTATGGCGTCAAT | Primer for amplification of insert in reporter plasmid pAMS187 |
| BA3946 | TTATGGCTGTATCATGTTATCGAACC | Primer for amplification of insert in reporter plasmid pAMS188 |
| BA3947 | CGATAAAGAGCGAAACCTGGAT | Primer for amplification of insert in reporter plasmid pAMS187 |
| BA3948 | AAAACCGTCTACAATCTCGTACTC | Primer for amplification of insert in reporter plasmid pAMS188 |
| BA3949 | TTAAGGAACCCTTTGTAAGTCAGG | Primer for amplification of insert in reporter plasmid pAMS201 |
| BA3950 | CTTGTCATGTTCAACAACGCGATATC | Primer for amplification of insert in reporter plasmid pAMS202 |
| BA3953 | TATTGGTTTCGCGAGTGATTAAATTA | Primer for amplification of insert in reporter plasmid pAMS205 |
| BA3960 | GAGCCCACGTTATGACCAGTAAC | Primer for amplification of insert in reporter plasmid pAMS201 |
| BA3961 | GGCTTAATACCGACCAGAGAAG | Primer for amplification of insert in reporter plasmid pAMS202 |
| BA3964 | ATTAATACTGAGAAATGATCTTCGCC | Primer for amplification of insert in reporter plasmid pAMS205 |
| BA3999 | CCAGGGAAAGATCAGATTATCGTATA | Primer for amplification of insert in reporter plasmid pAMS227 |
| BA4000 | CTTTCAGGATTTGCGACTGTTTTTC | Primer for amplification of insert in reporter plasmid pAMS227 |
| BA4001 | CATCATGTTATCGCCGATCATC | Primer for amplification of insert in reporter plasmid pAMS231 |
| BA4002 | GGATCGTTAAACAGATTGACCAGTTC | Primer for amplification of insert in reporter plasmid pAMS231 |
| BA4003 | CAATGCTAATGAATTCCCTACCCTA | Primer for amplification of insert in reporter plasmid pAMS228 |
| BA4004 | CTTCAAGAATATAGGCACGCTTGGTA | Primer for amplification of insert in reporter plasmid pAMS228 |
| BA4025 | CGATAGAAAAAGTTGAGGCGATTTTA | Primer for amplification of insert in reporter plasmid pAMS265 |
| BA4026 | GATAGTAATGCCAACGATGATGGAAG | Primer for amplification of insert in reporter plasmid pAMS265 |
| BA4031 | CTGATAAAAATGCGCTCAAGCTTA | Primer for amplification of insert in reporter plasmid pAMS291 |
| BA4032 | AAAAGTGGTAGCAGTTGAGATTTAAA | Primer for amplification of insert in reporter plasmid pAMS291 |
| BA4075 | TTCCACTGTCGGTTATAATAAAAACC | Primer for amplification of insert in reporter plasmid pAMS347 |
| BA4076 | GTTTGTAATGATGGATTCACCCATAA | Primer for amplification of insert in reporter plasmid pAMS347 |
| BA4077 | GCGTTTTCTCGGTCATTATTTGA | Primer for amplification of insert in reporter plasmid pAMS360 |
| BA4078 | CCTCCTCTTTCGACATCAATTCAG | Primer for amplification of insert in reporter plasmid pAMS360 |
| BA4079 | GTATTTCAGCTGGGCGTCATTG | Primer for amplification of insert in reporter plasmid pAMS361 |
| BA4080 | CTCTGATTACGTGCGGTCATCG | Primer for amplification of insert in reporter plasmid pAMS361 |
| BA4081 | GCCTCCGACAATTACTACTACC | Primer for amplification of insert in reporter plasmid pAMS362 |
| BA4082 | AGATACGGGTGTCATTGGCTAC | Primer for amplification of insert in reporter plasmid pAMS362 |
| BA4082 | AAAAAGAAGCTCAGCAATCCAC | Primer for amplification of insert in reporter plasmid pAMS363 |
| BA4084 | TTTGCGGGAAGAATGGAAATAATATT | Primer for amplification of insert in reporter plasmid pAMS363 |
| BA4085 | CAGTTTATCGTGCTGATGACTAC | Primer for amplification of insert in reporter plasmid pAMS364 |
| BA4086 | ATAAATTCTTTTGCCTGAATCGCATC | Primer for amplification of insert in reporter plasmid pAMS364 |
| BA4089 | AACTTCCACAGTTACCACTTAGC | Primer for amplification of insert in reporter plasmid pAMS366 |
| BA4090 | GACTGAAACTTCTTGATAAACAGGTT | Primer for amplification of insert in reporter plasmid pAMS366 |
| BA4091 | CAGGAAAAATTACGCCAAAAACTTC | Primer for amplification of insert in reporter plasmid pAMS367 |
| BA4092 | TAATAAACAGATTAAATACGCTGCCC | Primer for amplification of insert in reporter plasmid pAMS367 |
| BA4093 | GTTTCGCTGACCATCAACTCCC | Primer for amplification of insert in reporter plasmid pAMS368 |
| BA4094 | ATGTTCGACGGCATCACGAATG | Primer for amplification of insert in reporter plasmid pAMS368 |
